# An impaired ubiquitin-proteasome system increases APOBEC3A abundance

**DOI:** 10.1093/narcan/zcad058

**Published:** 2023-12-19

**Authors:** Margo Coxon, Madeline A Dennis, Alexandra Dananberg, Christopher D Collins, Hannah E Wilson, Jordyn Meekma, Marina I Savenkova, Daniel Ng, Chelsea A Osbron, Tony M Mertz, Alan G Goodman, Sascha H Duttke, John Maciejowski, Steven A Roberts

**Affiliations:** School of Molecular Biosciences, Washington State University, Pullman, WA 99164-7520, USA; School of Molecular Biosciences, Washington State University, Pullman, WA 99164-7520, USA; Molecular Biology Program, Sloan Kettering Institute, Memorial Sloan Kettering Cancer Center, New York, NY 10065, USA; School of Molecular Biosciences, Washington State University, Pullman, WA 99164-7520, USA; School of Molecular Biosciences, Washington State University, Pullman, WA 99164-7520, USA; School of Molecular Biosciences, Washington State University, Pullman, WA 99164-7520, USA; School of Molecular Biosciences, Washington State University, Pullman, WA 99164-7520, USA; School of Molecular Biosciences, Washington State University, Pullman, WA 99164-7520, USA; School of Molecular Biosciences, Washington State University, Pullman, WA 99164-7520, USA; School of Molecular Biosciences, Washington State University, Pullman, WA 99164-7520, USA; Department of Microbiology and Molecular Genetics, University of Vermont Cancer Center, University of Vermont, Burlington, VT 05405, USA; School of Molecular Biosciences, Washington State University, Pullman, WA 99164-7520, USA; School of Molecular Biosciences, Washington State University, Pullman, WA 99164-7520, USA; Molecular Biology Program, Sloan Kettering Institute, Memorial Sloan Kettering Cancer Center, New York, NY 10065, USA; School of Molecular Biosciences, Washington State University, Pullman, WA 99164-7520, USA; Department of Microbiology and Molecular Genetics, University of Vermont Cancer Center, University of Vermont, Burlington, VT 05405, USA

## Abstract

Apolipoprotein B messenger RNA (mRNA) editing enzyme, catalytic polypeptide-like (APOBEC) cytidine deaminases cause genetic instability during cancer development. Elevated APOBEC3A (A3A) levels result in APOBEC signature mutations; however, mechanisms regulating A3A abundance in breast cancer are unknown. Here, we show that dysregulating the ubiquitin-proteasome system with proteasome inhibitors, including Food and Drug Administration-approved anticancer drugs, increased A3A abundance in breast cancer and multiple myeloma cell lines. Unexpectedly, elevated A3A occurs via an ∼100-fold increase in A3A mRNA levels, indicating that proteasome inhibition triggers a transcriptional response as opposed to or in addition to blocking A3A degradation. This transcriptional regulation is mediated in part through FBXO22, a protein that functions in SKP1–cullin–F-box ubiquitin ligase complexes and becomes dysregulated during carcinogenesis. Proteasome inhibitors increased cellular cytidine deaminase activity, decreased cellular proliferation and increased genomic DNA damage in an A3A-dependent manner. Our findings suggest that proteasome dysfunction, either acquired during cancer development or induced therapeutically, could increase A3A-induced genetic heterogeneity and thereby influence therapeutic responses in patients.

## Graphical Abstract

**Graphical Abstract:**
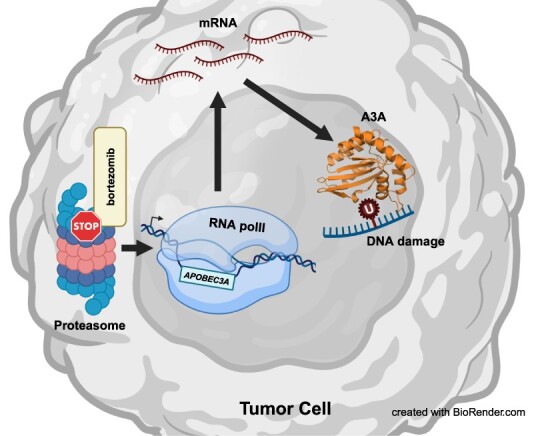
created with BioRender.com

## Introduction

Apolipoprotein B messenger RNA (mRNA) editing enzyme, catalytic polypeptide-like (APOBEC) cytidine deaminases are the second most common cause of mutations in sequenced human tumors (1). The APOBEC3 subfamily normally functions within the innate immune system to restrict viruses and transposons by creating mutations within foreign DNA (2). Off-target enzymatic activity generates cytidine to uridine modifications on genomic single-stranded DNA (ssDNA) formed primarily during DNA replication, and to a lesser extent during transcription or DNA repair (3–10). Such APOBEC signature mutations, characterized by C-to-T or C-to-G mutations within TCW motifs (W = A or T) (11) [also referred to as COSMIC single base substitution (SBS) signatures 2 and 13], are present in 30% of sequenced tumor samples from multiple cancer types (12), with the highest abundance observed in head and neck, breast, bladder, cervical and lung cancers (13).

Off-target APOBEC activity promotes tumorigenesis by causing oncogenic driver mutations such as *PIK3CA* E542K or *PIK3CA* E545K (14) and can contribute to therapeutic resistance to tamoxifen in estrogen receptor-positive breast cancer cells (15). Of the seven APOBEC3 proteins, we and others have provided substantial evidence that APOBEC3A (A3A) is a major contributor to APOBEC-induced mutations (16–18). We have shown that among the APOBEC3 genes, A3A expression is the most strongly correlated with APOBEC-induced mutation load in human cancers (17). A3A provides the majority of cytidine deaminase activity in whole cell extracts containing RNA [which inhibits APOBEC3B (A3B) activity] from a variety of breast cancer cell lines. An A3A favored mutation motif of YTCA to YTTA or YTGA is enriched in tumors with the highest load of APOBEC signature mutations (18). Finally, deletion of A3A from breast cancer and B-cell lymphoma cell lines nearly abolished accumulation of APOBEC signature mutations (i.e. SBS2 and SBS13) (16), directly implicating A3A in cancer mutagenesis.

Previous work on APOBEC dysregulation and its mutagenic activity in cancer cells has centered around APOBEC transcription and subcellular localization, where transcriptional activation and nuclear localization contribute to mutagenesis (19,20). Although A3A mRNA levels correlate positively with APOBEC signature mutation load (17), outlier samples in this association suggest that additional non-transcriptional mechanisms may also influence A3A protein abundance within cancer cells. During viral infection, multiple APOBEC members are regulated post-translationally by the ubiquitin-proteasome pathway, making this pathway a candidate for controlling A3A abundance in tumors (21). Human hepatocytes containing UBE2L3 polymorphisms have been found to be more susceptible to hepatitis B viral infection due to increased proteasome-mediated degradation of A3A (22). Additionally, multiple overexpressed APOBEC3 family members have been shown to be controlled by the activity of ubiquitin ligase complexes containing von Hippel–Lindau tumor suppressor protein (pVHL) and ariadne RING-in-between-RING E3 ubiquitin protein ligase 1 (ARIH1) (23).

The ubiquitin-proteasome pathway is frequently dysregulated during carcinogenesis and tumor growth (24,25). In many cases, changes to this pathway involve increased proteolytic activity and degradation of tumor suppressors, which promote tumorigenesis. Therefore, tumor samples often have higher levels of chymotrypsin-like proteasome activity compared to neighboring normal tissue (26). This is particularly true for breast cancers as most primary tumor samples exhibit upregulated proteasome activity (27). α-Estrogen receptor-negative breast carcinomas often acquire higher expression of the proteasome subunit and activator, LMP2 and PA700 (26), and *TP53* mutations upregulate proteasome activity by increasing proteasome subunit gene expression in triple-negative breast cancer (28). Additionally, increased expression of the E3 ubiquitin ligases, MDM2 and RING1, is a canonical oncogenic event leading to degradation of the tumor suppressor p53 (29). Depending on the component altered, the loss of ubiquitin-proteasome pathway function can also promote cancer initiation, metastases and drug resistance. pVHL itself is a tumor suppressor and germline inactivating mutations in this gene lead to a variety of kidney cancers presumably caused by inappropriate protein degradation (30). Moreover, reduced expression or mutations in F-box proteins that mediate substrate recognition for SKP–cullin E3 ubiquitin ligases can also promote tumorigenesis. Multiple cancer types acquire somatic mutations in the gene *FBXW7*, which encodes an F-box protein important for the degradation of tumor promoting proteins such as Cyclin E1, c-Myc, mTOR, Notch1 and SNAIL (31,32). Accordingly, loss of FBXW7 increases tumor incidence in mice. Similarly, reduced activity of the F-box protein, FBXO22, contributes to tumor progression and metastasis in lung and breast cancers by increasing levels of key oncogenic factors such as Bach1, HDM2 and SNAIL (33). Since APOBEC3 protein abundance has been shown to be controlled by proteasome activity in human embryonic kidney cells (23), somatically acquired alterations in such ubiquitin-proteasome pathway factors may similarly cause changes in A3A protein abundance leading to enhanced cancer cell mutagenesis.

Because of altered ubiquitin-proteasome activity acquired during tumorigenesis, treatment of cancers can also target changes in proteasomal degradation (25). The Food and Drug Administration has approved use of proteasome inhibitors (i.e. bortezomib, carfilzomib and ixazomib) for the treatment of multiple myeloma and mantle cell lymphoma, where they are used in the current standard care regimen. The efficacy of proteasome inhibitor treatment against multiple myeloma is due to a cellular dependence on the proteasome to compensate for the high production of immunoglobulins (34). The potential to expand the usage of proteasome inhibitors exists because other cancer types, such as basal-like triple-negative breast cancer, have been reported to be especially dependent on high proteasome activity (35). However, utilization of these therapies could have the unintended consequence of upregulating a major cancer mutator such as A3A.

Here, we investigate in breast and multiple myeloma cancer cell lines the impact of proteasome activity on A3A cellular abundance and activity. We show that treatment of a variety of cell types with the proteasome inhibitor, MG132, results in higher levels of both exogenous and endogenous A3A proteins. This effect is specific to A3A among APOBEC3 family members. The A3A elevated by proteasome inhibition is catalytically active, which is likely the cause of elevated nuclear DNA damage and slowed cellular proliferation. Surprisingly, the increase in A3A protein level is mirrored by a similar increase in A3A mRNA abundance indicating that inhibited proteasome function drives a transcriptional response regulating A3A. Knockdown of the ubiquitin-proteasome pathway component, FBXO22, results in a similar increase in A3A transcript and protein levels. Therapeutic proteasome inhibitors bortezomib, carfilzomib and ixazomib also increase A3A cytidine deaminase activity, transcripts and protein abundance in both breast cancer and multiple myeloma (the cancer type most commonly treated with clinical proteasome inhibitors) cell lines, indicating that clinical use of these drugs may unintendingly increase A3A-mediated DNA damage and mutation.

## Materials and methods

### Cell culture conditions

BT474, MDA-MB-453, MM.1S, U266B1 and RPE-1 cell lines were obtained from American Type Culture Collection (ATCC) and cultured in Hybri-Care medium + 10% fetal bovine serum (FBS), L-15 Leibovitz media + 10% FBS, RPMI 1640 + 10% FBS, RPMI 1640 + 15% FBS and Dulbecco’s modified Eagle medium + 10% FBS, respectively. BT474, RPE-1, MM.1S and U266B1 cells were incubated at 37°C with 5% CO_2_. MDA-MB-453 cells were incubated at 37°C with room CO_2_. MDA-MB-453 and BT474 cells transduced with lentiviral vectors expressing non-specific scramble small hairpin RNA (shRNA), A3A-targeting shRNA or A3B-targeting shRNA were described previously (17). The identity of all cell lines used in this study was authenticated by assessing the maintenance of published morphology characteristics throughout culturing. All multiple myeloma cells used for experiments were passaged <10 times from the original ATCC cryovial.

### Cloning for lentiviral shRNA plasmids and A3A expression plasmids

Oligos with shRNA sequences (listed in Supplementary Table S1) were phosphorylated with T4 PNK (NEB M0201S) and annealed. To create the lentiviral shRNA plasmids, the annealed oligos were cloned into the AgeI and EcoRI sites of plasmid pLKO.1 hygro (Addgene, #24150). HA-tagged A3A was expressed using a NEO-marked lentiviral vector, pTM-682 (Supplementary Data), which has a CMV promoter modified for reduced expression that also contains a pair of TETO sequences enabling doxycycline-induced expression in cells expressing TETR. Strep-tagged A3A mutants containing either a K137R mutation or full lysine to arginine substitutions (i.e. residues 30, 47, 60, 137 and 159) were created by modifying pTM-216 from (17). Geneblocks obtained from Integrated DNA Technologies containing K30/47/60R mutations, a K137R mutation or a K159R mutation were serially cloned into pTM-216 after polymerase chain reaction (PCR) amplification with oTM-58 and oTM-59 or 5′ phosphorylated oCC-101 and oCC-102 (only used to amplify the K159R mutation geneblock) and digesting with DraI and EagI, BglII and StuI, or StuI and AgeI, respectively. Wild-type (WT) or mutated A3A was transferred to NAT-marked Piggybac payload vector (named pTM-897) containing promoter and wPRE elements identical to pTM-637 in (17) using Gateway cloning methods. Accurate insertion of DNA for each cloning step of plasmid construction was verified via Sanger sequencing.

### Transfections

Lentiviruses were generated via co-transfection of lentiviral plasmids and packaging plasmids psPAX2 (Addgene, #12260) and pMD2.G (Addgene, #12259) into either HEK293T cells (for shRNA plasmids) or HEK293T-TETR (17) (for pTM-682). The lentiviral supernatant was collected at 48 h post-transfection and added to the target cells along with Lentiblast transduction reagent (OZ Biosciences), which was diluted 1:250 into lentiviral supernatant. The lentiviral supernatant was removed 24 h post-transduction. For cell lines with shRNA sequences expressed from pLKO.1 hygro, stable cell populations were selected 72 h post-transduction with hygromycin B at 200 μg/ml. For RPE-1 cells expressing HA-tagged A3A from pTM-682, stable cell populations were selected 72 h post-transduction with G418 at 400 μg/ml. RPE-1 cells were transiently transfected to express strep-tagged constructs of WT A3A, A3A K137R or an A3A mutant with all lysines converted to arginine. Cells were harvested 72 h post-transfection without NTC selection for generating RNA extracts and whole cell lysate.

### Cell treatments

Cell lines were treated with proteasome inhibitors diluted in dimethyl sulfoxide (DMSO), cycloheximide in DMSO or treated with a DMSO control and incubated for 3, 6, 12 or 24 h in normal culturing conditions before harvesting cell pellets. Cell pellets were lysed in M-PER lysis buffer (Thermo Fisher Scientific) containing Pierce Protease and Phosphatase Inhibitor Mini Tablets (Thermo Fisher Scientific) for activity and western analysis (unless stated otherwise) or saved for RNA assays.

### Immunoblots

GAPDH, tubulin, HA-tagged A3A and strep-tagged A3A immunoblotting: 25–30 μg cell extract in M-PER (quantified via Bradford assay) was run on pre-made Mini-Protean TGX gels (Bio-Rad) in 1× TGS buffer [25 mM Tris, 250 mM glycine, 0.1% sodium dodecyl sulfate (SDS)]. Then, protein was transferred onto a 0.2 μm polyvinylidene fluoride membrane (Thermo Scientific) via a Trans-Blot turbo transfer system (Bio-Rad) in transfer buffer (25 mM Tris, 250 mM glycine, 20% methanol) using the ‘low MW’ or ‘mixed MW’ settings as appropriate. Membranes were blocked in 2% ECL prime blocking agent (Cytiva) in TBST (20 mM Tris, 150 mM NaCl, 0.1% Tween 20). Membranes were incubated at 4°C in Rb-anti-GAPDH (Proteintech 10494-1-AP; 1:20000), Rb-anti-HA (Abcam ab9110; 1:15000), Rb-anti-alpha tubulin (Abcam ab4074, diluted 1:20000) or Rb-anti-strep (Abcam ab76949, diluted 1:10000) diluted in 1% ECL in TBST at 4°C or room temperature for 12–18 h. Blots were incubated in anti-Rb IgG HRP (Abcam ab97051; 1:20000) in 1% ECL at 4°C for 1.5 h. Blots were developed with ECL prime western blotting detection reagent (Cytiva) before imaging on a Bio-Rad ChemiDoc MP Imaging System using the ‘chemi’ auto exposure or signal accumulation settings.

For endogenous A3A immunoblotting, cells were lysed in RIPA buffer (150 mM NaCl, 50 mM Tris–HCl, pH 8.0, 1% NP-40, 0.5% sodium deoxycholate, 0.1% SDS, Pierce Protease Inhibitor Tablet, EDTA-free). Quantification of RIPA extracts was performed using the Thermo Fisher Scientific Pierce BCA Protein Assay Kit. Protein transfer was performed by wet transfer using Towbin buffer (25 mM Tris, 192 mM glycine, 0.01% SDS, 20% methanol) and nitrocellulose membrane. Blocking was performed in 5% milk in TBST (19 mM Tris, 137 mM NaCl, 2.7 mM KCl and 0.1% Tween 20) for 1 h at room temperature. The following antibodies were diluted in 1% milk in TBST: anti-A3A (Maciejowski Lab; 01D05; western blot, 1:500), anti-tubulin (Abcam; ab8224; western blot, 1:3000) and anti-mouse IgG HRP (Thermo Fisher Scientific; 31432; 1:10000).

Blots were analyzed in Bio-Rad Image Lab using the ‘volume tools’ setting to calculate band intensity. Local background subtraction was used for band analysis except in GAPDH blots of RPE-1 cells treated with cycloheximide and MG132, where background subtraction from a chosen representative blot area was used. In HA-A3A and GAPDH blots of RPE-1 cells treated with cycloheximide and MG132, band volumes on each blot were normalized to one sample band before relative HA-A3A to GAPDH signal intensity was calculated for every sample.

### Cytidine deaminase assays

Cytidine deaminase assays were performed with the hairpin-forming oligonucleotide substrate oTM-814 Cy5. Five micrograms of cell extract (quantified by Bradford assay) was used to assess deaminase activity. Reactions containing cell extract with 20 mM Tris–HCl, pH 7.5, 1 mM DTT, 1 mM EDTA and 1.105 μM oTM-814 Cy5 in 20 μl were incubated for 8–24 h (as indicated) at 37°C and then stopped by addition of 1 μl Proteinase K (0.07 mg/ml final) and Proteinase K buffer (10 mM Tris–HCl, pH 8, 1 mM EDTA, 0.5% SDS final). Reactions were incubated at 37°C for 20 min for proteinase digestion. One molar NaOH (0.1 M final) and formamide buffer (47.5% formamide, 9 mM EDTA, 0.0125% SDS final) were added and samples were incubated at 95°C for 10 min to cleave abasic sites and then run on a pre-warmed 15% polyacrylamide gel with 7.9 M urea in 1× TBE. Gels were imaged on a ChemiDoc MP using the Cy5 imaging settings.

### Measuring gene expression via qRT-PCR

Cells were harvested during the log phase of growth and put through a homogenizer column and a total RNA kit (Omega Biotek). Complementary DNA (cDNA) was generated by combining 2 μg of DNase I-treated (Thermo Fisher) RNA with the cDNA reaction mixture (2.5 μM oligo dT23VN and 3.5 μM random hexamers), 0.5 mM dNTPs, Mashup RT Reaction Buffer (25 mM Tris–HCl, pH 8.3, 25 mM MOPS, pH 7.9, 60 mM KCl, 4 mM MgCl_2_, 5% glycerol, 0.006% IGEPAL CA-630), 10 mM DTT, Mashup Reverse Transcriptase and 16 U RNase Inhibitor (RiboLock RNase Inhibitor, Thermo Fisher) in a total volume of 20 μl. The reactions were incubated at 25°C for 5 min, 42°C for 60 min and finally 65°C for 20 min, and then stored at −20°C. The quantitative reverse transcription PCR (qRT-PCR) reaction was made by combining 2 μl of cDNA template with 10 μl of Forget-Me-Not Eva-Green 2× Master Mix, 0.5 μl of 10 μM primer and 7 μl of sterile water for a total reaction volume of 20 μl. The reactions were run on a Bio-Rad CFX96 machine with the following protocol: incubate at 95°C for 5 min and then put through 40 cycles of 95°C for 10 s and 62.5°C for 1 min. Raw Cq values for all experiments are provided in Supplementary Table S2. Melt curves were used to confirm that singular products were generated for all reactions.

### csRNA-seq library preparation

Capped small RNA sequencing (csRNA-seq) was performed as described in (36) with modified 5′ enrichment as detailed below. Small RNAs of ∼20–60 nucleotides were size selected from 0.4–3 μg of total RNA by denaturing gel electrophoresis. A 10% input sample was set aside and the remainder enriched for 5′-capped RNAs. Monophosphorylated RNAs were selectively degraded by 1 h incubation with Terminator 5′-Phosphate-Dependent Exonuclease (Lucigen). Subsequently, RNAs were 5′ dephosphorylated with thermostable QuickCIP (NEB) by heating samples to 75°C for 30 s and then chilling them on ice for 60 min. Then samples were incubated at 37°C for another 30 min (36). Input (sRNA) and csRNA-seq libraries were prepared as described in (37) using RppH (NEB) and the NEBNext Small RNA Library Prep Kit, and amplified for 12 cycles.

### csRNA-seq data analysis

Transcription start regions (TSRs), transcription start sites and their activity levels were determined by csRNA-seq and analyzed using HOMER v4.12 (38). Additional information, including analysis tutorials, is available at https://homer.ucsd.edu/homer/ngs/csRNAseq/index.html. TSR files for each experiment were added to Gene Expression Omnibus (GEO) data. csRNA-seq and total small RNA-seq (input) were analyzed as detailed in the HOMER csRNA-seq tutorial (http://homer.ucsd.edu/homer/ngs/csRNAseq/). Sequencing reads were trimmed of their adapter sequences using HOMER (‘batchParallel.pl “homerTools trim -3 AGATCGGAAGAGCACACGTCT -mis 2 -minMatchLength 4 -min 20” none -f {csRNA_fastq_path}/*fastq.gz’) and aligned to the appropriate genome using STAR (39): *STAR --genomeDir GenomeIndexForhg38/ --runThreadN 24 --readFilesIn exp1.csRNA.fastq.gz.trimmed --outFileNamePrefix exp1.csRNA.fastq.gz.trimmed. --outSAMstrandField intronMotif --outMultimapperOrder Random --outSAMmultNmax 1 --outFilterMultimapNmax 10000 --limitOutSAMoneReadBytes 10000000*. Tag directories were generated (*makeTagDirectory csRNA-tagDir/ csRNA.fastq.gz.trimmed.Aligned.out.sam -genome hg38.genome.fa -checkGC -fragLength 150*) and peaks called using findcsRNATSS.pl (*findcsRNATSS.pl csRNA-TagDir/ -o outputPrefix -i input-TagDir/ -gtf genes.gtf -genome genome.fasta*) (38). Differential expressed loci were defined using HOMER getdiffexpression.pl (log_2_ fold, false discovery rate 0.05) and motifs using findMotifsGenome.pl (http://homer.ucsd.edu/homer/motif/) (40).

### Cell growth assays

MDA-MB-453 cells were counted with a K2 Cellometer (Nexcelom) and plated at 5000 live cells/well in a six-well tissue culture treated plate (Fisher Scientific) 24 h before treatment with a final concentration of 0.1 or 0.5 μM MG132 diluted in DMSO or a DMSO control. Twenty-four hours after treatment, cells were counted to assess cell number using the cell analysis program by bright-field imaging on a Cytation5 and counted every 3 days thereafter until 21 days post-plating. Media and MG132 or DMSO were refreshed every 3 days after imaging.

### Immunofluorescence

MDA-MB-453 or BT474 cells were plated on poly-lysine-coated coverslips and treated with bortezomib (13 nM for 6 h), DMSO (1 μl/ml for 24 h) or zeocin (100 μg/ml for 24 h). To identify cycling cells, EdU was added to culture media to a final concentration of 10 μM for 2 h. Cells were fixed with 2% paraformaldehyde for 15 min at room temperature followed by 0.5% Triton X-100 permeabilization for 5 min. Then, EdU staining was performed by using Click-iT EdU Alexa Fluor 488 Imaging Kits (Invitrogen, C10337) according to the manufacturer’s instructions. γH2AX was stained with anti-γH2AX antibodies (EMD Millipore, 05-636-1, 1:1000) for 2 h at room temperature followed by anti-mouse secondary antibody Alexa Fluor 647 (Invitrogen, A21235). Cells were stained with Hoechst (1 μg/μl) and mounted with Prolong Gold Antifade Reagent (Invitrogen, P36934).

Images were acquired on the Nikon Eclipse Ti2-E equipped with a CSU-W1 spinning disk. All of the images were processed by maximal projection of the z stack image series and analyzed by Fiji. After separating channels using the ImageJ Macro Batch Split Channels tool, nuclear masks were generated by Fiji Macro CLAIRE, whereby nuclei are identified by radius in the Hoechst channel, binary processed (filling holes and watershed) and applied with auto local threshold (Phansalkar). Nuclear EdU and Hoechst intensity values were collected by measuring the mean intensity within nuclear masks (region of interest measurement). To identify γH2AX foci, images were processed with background subtraction and Gaussian blur. γH2AX foci were displayed in ‘find maximum’ with output ‘point selection’ with manually adjusted parameters. The number of nuclear γH2AX foci was calculated by dividing the total γH2AX intensity at the displayed points (within the nuclear masks) with the intensity of a single γH2AX focus. All ImageJ macro and R codes were shared by M. Ferrari (M. Jasin Laboratory, Memorial Sloan Kettering Cancer Center).

### Statistical analysis

All statistical analyses were performed in GraphPad Prism (version 9.5.0). At least three biological replicates were analyzed for each experimental condition tested. Gene expression changes were analyzed by ratio paired *t*-tests. Cytidine deaminase activity and fold changes of cell growth comparisons were made by unpaired *t*-tests. Number of foci in immunofluorescence experiments were compared by paired *t*-tests.

## Results

### Proteasome inhibitor MG132 increases exogenous A3A in RPE-1 cells

**Figure 1.**
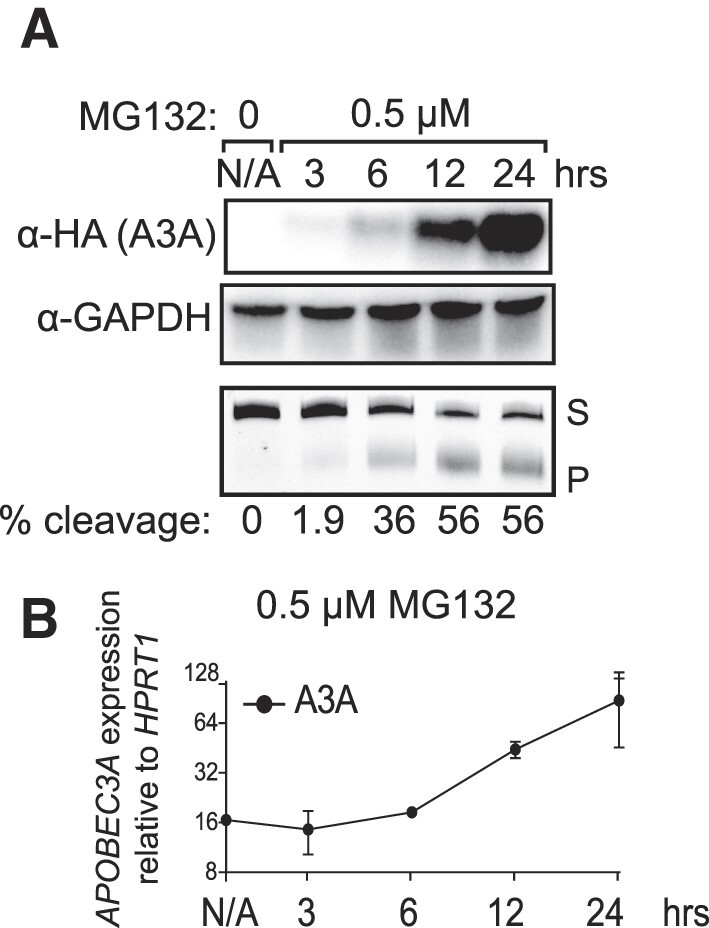
A3A is increased with inhibition of the UPS by MG132 in RPE-1 cells. (**A**) RPE-1 cells that had been transduced with HA-tagged A3A were treated with 0.5 μM MG132 and samples were collected at various time points, for a total of 24 h. Western blot analysis allowed quantification of A3A at all time points via an anti-HA antibody and GAPDH was used to ensure equal loading. An *in vitro* assay involving incubation of whole cell extracts with a hairpin substrate was used to determine cytidine deaminase activity by A3A in the form of percent cleavage. (**B**) RPE-1 cells treated with 0.5 μM MG132 were used to assess A3A mRNA levels for various time points via qRT-PCR. *APOBEC3A* expression was quantified relative to *HPRT1* levels. S, substrate; P, product.

To investigate the potential proteasomal regulation of A3A, we treated immortalized RPE-1 cells overexpressing C-terminally HA-tagged catalytically active A3A with the proteasome inhibitor MG132 and assessed A3A abundance by western blot. Within 3 h of 0.5 μM MG132 treatment, A3A protein levels began to increase and continued to increase through 24 h (Figure 1A), where A3A protein levels were >40-fold higher than pre-treatment levels. As the increased A3A could result from the stabilization of unfolded protein, we subsequently utilized an *in vitro* cytidine deaminase assay (17) to assess whether the additional A3A was catalytically active. We incubated whole cell extracts containing A3A with a 5′ Cy5-labeled hairpin-forming oligonucleotide with a single A3A target cytidine (in a TCA motif) present in the 4-bp loop, and measured substrate fragmentation as a proxy for deaminase activity. After a 3 h incubation, extracts from untreated cells produced low levels of substrate fragmentation (2% cleavage); however, with increasing time in the presence of MG132, the percentage of substrate cleaved increased 27.5-fold (55% cleavage) by 24 h (Figure 1A, bottom panel), indicating that the vast majority of A3A induced by proteasome inhibition is catalytically active. We also harvested total RNA from MG132-treated RPE-1 cells at various times following the addition of MG132 to media and evaluated A3A transcript abundance via qRT-PCR. Transcript levels for the exogenously expressed A3A construct increased ∼4-fold over the 24 h time course (Figure 1B). Elevation of A3A mRNA lagged behind that of A3A protein abundance through MG132 treatment, suggesting that the initial increase is mediated by lack of protein degradation. However, we cannot exclude that some of the increase in A3A protein is due to stabilization of A3A transcript.

### MG132 increases endogenous A3A in multiple BRCA cell lines

**Figure 2.**
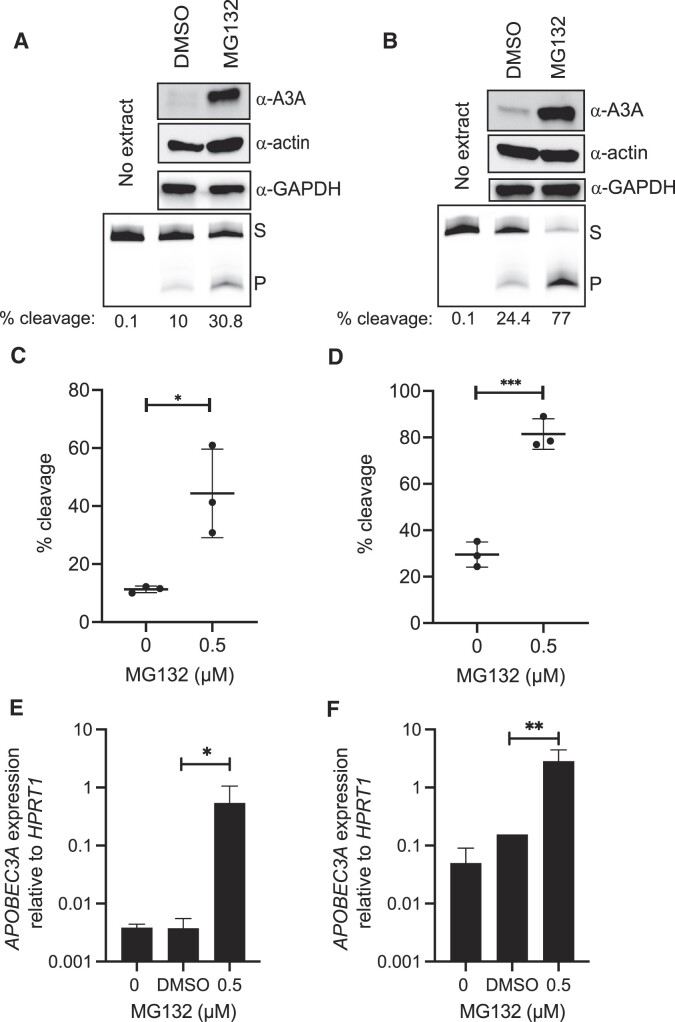
A3A is increased with inhibition of the UPS by MG132 in BRCA cells. A3A abundance and activity were measured after 24 h treatment with 0.5 μM MG132 in MDA-MB-453 (**A**, **C**, **E**) and BT474 cells (**B**, **D**, **F**). Representative images of western blot GAPDH analysis and denaturing gels for A3A substrate cleavage (A, B). A3A activity was determined with the *in vitro* cytidine deaminase assay and percent cleavage was calculated and graphed (*n* = 3). Percent substrate cleaved of untreated and treated MDA-MB-453 (C) and BT474 (D) cells. Significant changes are represented by asterisks to denote *P*-values of unpaired *t*-tests comparing MG132 to the DMSO control. Error bars represent one standard deviation. The transcript levels for A3A, when compared to *HPRT1*, increased significantly by (E) 145-fold (ratio paired *t*-test; *P*-value 0.0338) and (F) 18-fold (ratio paired *t*-test; *P*-value 0.0019). S, substrate; P, product. *P ≤ 0.05, **P ≤ 0.01 and ***P ≤ 0.001.

We next sought to determine whether proteasome inhibition causes similar effects on endogenous A3A in breast cancer cell lines. We evaluated the impact of MG132 on two different BRCA cell lines, BT474 and MDA-MB-453, which have previously been shown to have ongoing APOBEC-induced mutagenesis (16,41). After 24 h treatments with 0.5 μM MG132, cells were harvested for western blot analysis, cytidine deaminase activity assays and A3A transcript level quantifications. As observed for overexpressed A3A in RPE-1 cells, A3A protein levels (as measured via western blot) in breast cancer cell lines MDA-MB-453 and BT474 dramatically increased in the presence of MG132 (Figure 2A and B). In accordance with protein levels, cellular cytidine deaminase activity increased 3.9- and 2.7-fold (Figure 2C and D) in MDA-MB-453 and BT474 cells, respectively (all unpaired *t*-test *P*-values <0.03), indicating that the increased endogenous A3A is catalytically active. MG132 binds to β subunits of the 20S proteasome and blocks 26S proteasome activity (42). Therefore, we expected the increase in protein levels, and by extension cytidine deaminase activity, to originate primarily from a lack of protein degradation in the presence of the proteasome inhibitor. Interestingly, we found that 0.5 μM MG132 treatment elevated A3A transcript levels 145-fold in MDA-MB-453 cells and 18-fold in BT474 cells (Figure 2E and F). This result indicates that the major impact of MG132 on A3A abundance is mediated through elevated transcript levels. Proteasome inhibition reportedly can influence mRNA abundance in yeast (43) and breast cancer cells (44,45); however, the mechanisms producing this effect are still unclear.

To determine whether MG132 primarily elevates A3A levels via increased mRNA abundance or decreased proteasomal degradation, we treated RPE-1 cells overexpressing C-terminally HA-tagged A3A independently or in combination with the translation inhibitor cycloheximide and MG132 for 6, 12 and 24 h. Western blot analysis of A3A protein levels in these cells revealed a significant increase in A3A abundance only with independent MG132 treatment, whereas combining cycloheximide with MG132 blocked induction of A3A (Supplementary Figure S1). This result demonstrates that increased A3A caused by MG132 treatment requires new translation of A3A mRNA as opposed to drug-induced inhibited degradation of existing A3A. Further supporting a lack of direct proteasomal degradation of A3A in RPE-1 cells, we transfected these cells with expression constructs for either C-terminally strep-tagged A3A (i.e. wild type) or an A3A-K137R mutant. K137 was previously identified as an A3A ubiquitination site in HEK293 cells (22). Despite removing the likely ubiquitination site, A3A K137R displayed similar protein abundances (relative to mRNA transcript) to WT A3A in RPE-1 cells (Supplementary Figure S1C), indicating that K137 does not significantly mediate A3A degradation. Moreover, exogenous expression of a non-ubquitinylatable A3A-5K>R mutant, where all five lysines in A3A were mutated to arginine, also resulted in similar A3A protein levels to WT A3A, suggesting that protein degradation does not significantly alter overexpressed A3A protein abundance. Thus, proteasome inhibition largely elevates A3A protein level by increasing A3A mRNA abundance.

**Figure 3.**
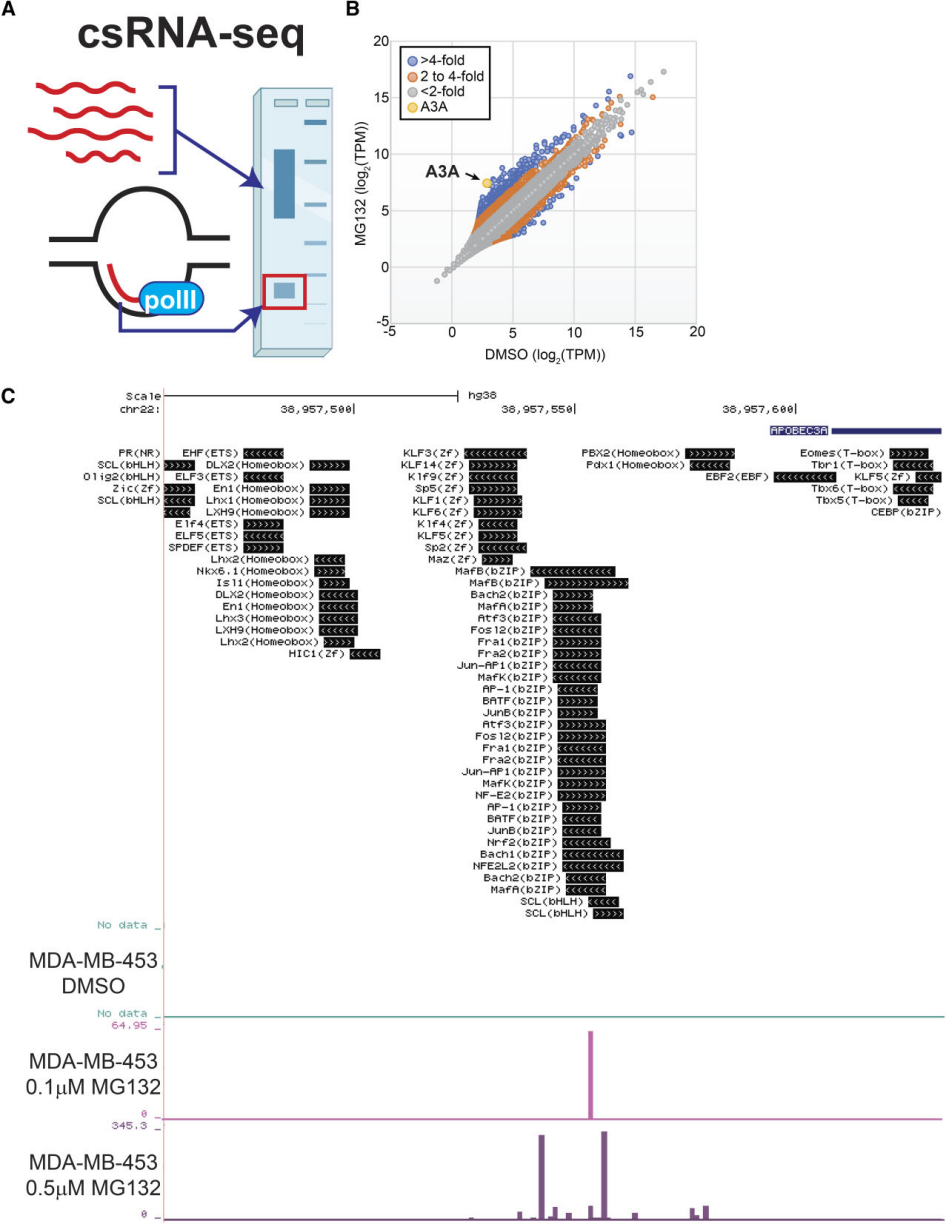
Nascent A3A transcription is increased upon proteasome inhibition. (**A**) Schematic of csRNA-seq methodology that captures actively initiating or ‘nascent’ transcription. Created with BioRender.com. (**B**) Comparison of csRNA-seq reads per gene promoter region (quantified in tags per million (TPM)) in DMSO-treated or 0.5 μM MG132-treated MDA-MB-453 cells. Dots indicate transcribed regulatory elements, such as promoters or putative enhancers. (**C**) UCSC data browser image of csRNA-seq reads aligning to the A3A promoter region following DMSO, 0.1 μM MG132 or 0.5 μM MG132 treatment. Known transcription factor binding sites proximal to the transcription start sites of the A3A transcripts are displayed.

Proteasome inhibition could elevate A3A mRNA by increasing transcript stability or by promoting new transcription of the *APOBEC3A* gene. To differentiate between these possibilities, we captured actively initiating or ‘nascent’ transcription initiation by csRNA-seq (38). Analysis of MDA-MB-453 cells treated with DMSO, 0.1 μM MG132 or 0.5 μM MG132 for 24 h revealed increased transcription initiation of the *APOBEC3A* gene in a dose-dependent manner (Figure 3). After 24 h in the presence of 0.5 μM MG132, nascent A3A mRNA levels increased 23-fold, the second greatest fold change of any gene observed in the dataset, indicating that MG132 treatment drives new A3A transcription (Supplementary Table S3).

**Figure 4.**
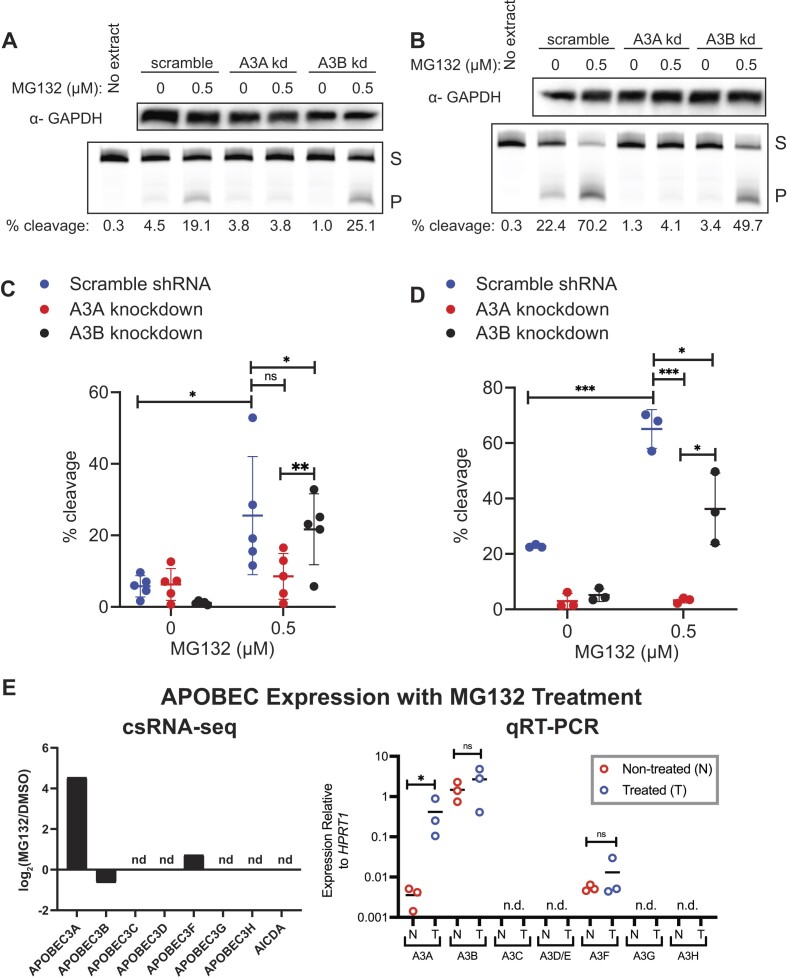
MG132 inhibition increases A3A specifically. Breast cancer cell lines MDA-MB-453 and BT474 were transduced with a non-specific scramble shRNA, A3A-targeting shRNA or A3B-targeting shRNA. Efficiency of shRNA knockdown lines previously published in (17). Representative images of western blot GAPDH analysis and denaturing gel of activity assays of the knockdown lines in MDA-MB-453 cells (**A**) or BT474 cells (**B**) treated with MG132 or DMSO control. Plotted percent substrate cleavage for the scramble shRNA, A3A shRNA and A3B shRNA expressing lines in MDA-MB-453 (**C**, *n* = 5) and BT474 (**D**, *n* = 3). Significant differences in A3A activity between the knockdown lines are marked with asterisks as compared by unpaired *t*-test. Mean is shown for all replicates and error bars represent one standard deviation. (**E**) Log_2_ fold change in transcription initiation of APOBEC3 family genes and *ACIDA* during a 24 h treatment of MDA-MB-453 cells with 0.5 μM MG132 compared to DMSO control. nd, not detected. Expression relative to *HPRT1* was quantified via qRT-PCR for all seven APOBEC3 proteins; only *APOBEC3A* showed a significant increase in expression after 24 h treatment with 0.5 μM MG132 (ratio paired *t*-test; *P*-value 0.0266). nd, not detected; S, substrate; P, product. *P ≤ 0.05 and ***P ≤ 0.001; ns, non-significant with *P*-value >0.05.

Next, we assessed whether MG132 induction was shared among APOBEC3 family members or specific to A3A. We utilized previously characterized versions of BT474 and MDA-MB-453 cells lentivirally transduced to express A3A-targeting, A3B-targeting or scramble control shRNAs (17) and measured cytidine deaminase activity 24 h post-treatment with MG132 or DMSO. MG132 treatment increased activity in the A3B- and scramble-targeting shRNA knockdown lines in MDA-MB-453 cells by 19.7- and 4.4-fold changes in percent substrate cleaved, respectively (all *P*-values ≤0.03); however, no elevation was observed in the A3A-targeting shRNA knockdown line (Figure 4A and C) suggesting that A3A specifically is responsible for the increased activity observed after proteasome inhibition. Similar results were seen in the other BRCA line tested, BT474, with fold changes of 2.8 and 7 in activity of the scramble- and A3B-targeting knockdown lines, respectively (*P*-values <0.02), with no significant increase in activity in the A3A-targeting knockdown line (Figure 4B and D).

Because the increase in A3A upon proteasome inhibition appears to be driven largely by elevated transcript levels, we also assessed the impact of MG132 treatment on the transcript abundance of all seven APOBEC3 family members. csRNA-seq analysis of each APOBEC3 gene and *AICDA* indicated that in MDA-MB-453 cells only *APOBEC3A*, *APOBEC3B* and *APOBEC3F* genes produced detectable nascent transcript and *APOBEC3A* was unique in its transcriptional induction by MG132 (Figure 4E). Steady-state transcript abundance mirrored these results. qRT-PCR measurement of APOBEC3 family members in MDA-MB-453 cells in the presence and absence of MG132 only detected A3A, A3B and A3F mRNA, with A3A transcript uniquely upregulated by MG132 (Figure 4E) providing further support of a transcriptional regulatory mechanism unique to A3A among APOBEC3 family members that is impacted by proteasome inhibition.

### A3A increases in an FBXO22-dependent manner

A3A transcript levels in peripheral blood cells, immortalized breast epithelial cells, and head and neck cancer cell lines can be induced through either JAK/STAT signaling or NF-κB binding to the A3A promoter region (46,47). Additionally, our csRNA-seq data indicate that Rel-A binding sites are enriched in the promoter regions of genes with nascent transcripts increased by MG132 (Supplementary Figure S2A). We therefore wondered whether proteasome inhibition might increase A3A transcription by activating one of these two pathways. We established MDA-MB-453 cell populations expressing STAT1, STAT2, REL-A or nontargeting shRNAs and treated them with MG132 or DMSO. Despite respective 82%, 70% and 95% reductions in transcript for each target gene (Supplementary Figure S3), MG132 treatment still produced a dramatic elevation of A3A transcript level as determined by qRT-PCR (Supplementary Figure S2B), indicating that proteasome inhibition induces A3A mRNA abundance through a novel mechanism, possibly by influencing Ets or bZIP family transcription factors whose binding sites are directly upstream of the A3A transcription start site (Figure 3C).

**Figure 5.**
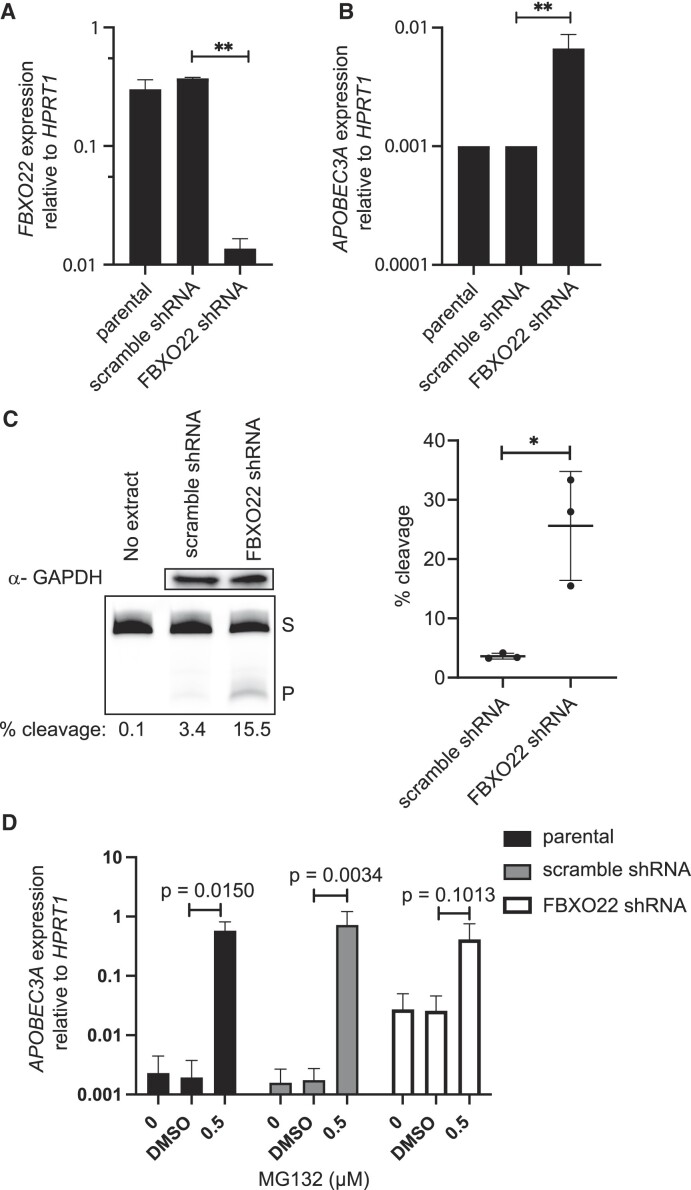
A3A increase is FBXO22 dependent. FBXO22 shRNA knockdown in MDA-MB-453 cells. (**A**) FBXO22 expression was reduced 30-fold (ratio paired *t*-test; *P*-value 0.0011) leading to (**B**) a 6.7-fold increase in A3A (ratio paired *t*-test; *P*-value 0.0086). (**C**) Representative images of GAPDH analysis by western blot and denaturing gel of A3A activity assays with MDA-MB-453 cells expressing a scramble shRNA or FBXO22 shRNA knockdown. Percent cleavage of hairpin substrate in activity assays plotted produced significant change from scramble to FBXO22 knockdown (*n* = 3). Mean shown for the replicates and error bars represent one standard deviation. (**D**) A3A expression relative to *HPRT1* was assessed in the parental, scramble shRNA and FBXO22 shRNA cell lines upon 24 h treatment with 0.5 μM MG132 (*n* = 3 independent measurements). The parental and scramble shRNA lines had a 296-fold increase (ratio paired *t*-test; *P*-value 0.0150) and 416-fold increase (ratio paired *t*-test; *P*-value 0.0034), respectively. FBXO22 shRNA did not have a significant increase in A3A (ratio paired *t*-test; *P*-value 0.1013). S, substrate; P, product. *P ≤ 0.05 and **P ≤ 0.01; ns, non-significant with *P*-value >0.05.

This mechanism likely involves factors targeted for proteasome destruction by an ubiquitin ligase containing FBXO22. FBXO22 is an F-box protein that binds substrates for Cullin–RING E3 ligases and targets them for destruction (48,49). FBXO22 is frequently dysregulated during cancer development (50) and has both tumor promoting and suppressive roles by targeting proteins like p53 (51) and PTEN for proteasome-mediated destruction (52). We expressed shRNAs targeting FBXO22 in MDA-MB-453 cells to reduce its expression (Figure 5A) as a genetic means of dysregulating the ubiquitin-proteasome pathway. shRNA-mediated reduction of FBXO22 expression 30-fold resulted in a 6.7-fold increase in A3A mRNA abundance (Figure 5B, ratio paired *t*-test *P*-value 0.0086) and a 7-fold increase in cytidine deaminase activity compared to scramble shRNA cells (Figure 5C, unpaired *t*-test *P*-value 0.0144), phenocopying the impacts of MG132 treatment. To determine whether FBXO22 depletion elevated A3A through the same pathway as MG132 treatment, we measured A3A transcript abundance in cell populations experiencing both FBXO22-targeting shRNA expression and MG132 treatment. The parental, scramble shRNA control and FBXO22 knockdown lines were each treated with 0.5 μM MG132 or DMSO for 24 h, after which A3A transcript levels were measured by qRT-PCR (Figure 5D). As expected, in the parental and scramble shRNA lines, A3A transcript levels increased 296- and 416-fold, respectively, upon MG132 treatment. The FBXO22 knockdown elevated A3A mRNA 15-fold in the absence of MG132. Combined FBXO22 knockdown and MG132 treatment, however, produced an additional 16-fold increase in A3A transcript to a level similar to treatment with the proteasome inhibitor alone. This apparent epistatic relationship is consistent with FBXO22 functioning in the same pathway as overall proteasome inhibition for augmenting A3A mRNA abundance. The relative difference in the extent of A3A induction between FBXO22 depletion and MG132 treatment may reflect either an incomplete reduction of FBXO22 activity or proteasome inhibition impacting A3A expression via multiple pathways/proteins, one of which is mediated through FBXO22.

### Clinical proteasome inhibitors induce A3A

**Figure 6.**
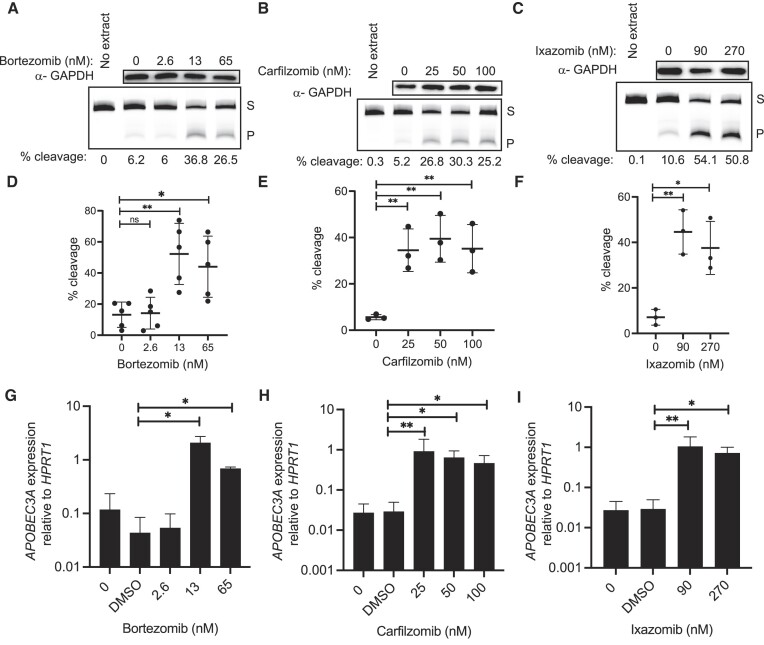
Other UPS inhibitors increase A3A. A3A activity, protein and transcript in BT474 cells after 24 h treatment with clinical proteasome inhibitors. Representative images of western blot GAPDH analysis and denaturing gel for cytidine deaminase assay with percent cleavage shown for (**A**) bortezomib, (**B**) carfilzomib and (**C**) ixazomib. Quantification of percent cleavage for all replicates of (**D**) bortezomib (*n* = 5), (**E**) carfilzomib (*n* = 3) and (**F**) ixazomib (*n* = 3) treated cells. Percent cleavage of activity assays for all replicates of treated cells showed significant differences in activity compared to control, as represented in *P*-values from unpaired *t*-test results. Panels (D)–(F) show mean of all replicates; error bars represent one standard deviation. (**G**–**I**) A3A expression relative to HPRT1 was evaluated via qRT-PCR. (G) Bortezomib at concentrations of 13 and 65 nM increased A3A 47.7-fold (ratio paired *t*-test; *P*-value 0.0106) and 15.8-fold (ratio paired *t*-test; *P*-value 0.0236), respectively. (H) Carfilzomib at concentrations of 25, 50 and 100 nM increased A3A 31.2-fold (ratio paired *t*-test; *P*-value 0.0075), 21.9-fold (ratio paired *t*-test; *P*-value 0.0100) and 16-fold (ratio paired *t*-test; *P*-value 0.0124), respectively. (I) Ixazomib at the concentrations of 90 and 270 nM increased A3A 35.8-fold (ratio paired *t*-test; *P*-value 0.0051) and 24.7-fold (ratio paired *t*-test; *P*-value 0.0324), respectively. S, substrate; P, product. *P ≤ 0.05 and **P ≤ 0.01; ns, non-significant with *P*-value >0.05.

Targeting the proteasome has provided therapeutic benefit for the treatment of cancer, particularly chemotherapy refractory multiple myeloma. This has led to the implementation of the proteasome inhibitor, bortezomib, often in combination with other agents, to treat this disease. Bortezomib is a boronic acid-based inhibitor that was synthesized to have a similar, but more targeted effect than MG132. Both inhibit the β5 subunit of the 20S proteasome, with bortezomib being a more potent and selective proteasome inhibitor (53). We tested a range of concentrations encompassing the IC50s (obtained from Selleckchem.com) of bortezomib on BRCA cell lines to determine whether they produced a similar increase in A3A levels to MG132. We found that in BT474 cells bortezomib increased A3A activity 3.97- and 3.34-fold at 13 and 65 nM, respectively, compared to DMSO treatment (Figure 6A and D) (all *P*-values <0.02). Unfortunately, many patients develop resistance to bortezomib, promoting a need for equally potent second-generation proteasome inhibitors. Carfilzomib and ixazomib have emerged as the two primary candidates for replacing bortezomib (54). We tested both second-generation inhibitors and similar results were observed with carfilzomib, where all concentrations tested (25, 50 and 100 nM) produced an average fold change of 6.5 in percent substrate cleaved after 24 h in BT474 cells (Figure 6B and E; all *P*-values <0.009). Ixazomib treatment confirmed the trend of increased A3A activity after proteasome inhibition with a maximum fold change in substrate cleavage compared to DMSO at 90 nM (Figure 6C and F), which remained elevated at 270 nM (all *P*-values <0.02).

In the BRCA line MDA-MB-453, 24 h treatments with proteasome inhibitors yielded similar results of elevated A3A activity compared to DMSO. Treatment with bortezomib produced a maximum fold change of 7.7 in substrate cleavage at 13 nM and remained elevated at 65 nM (*P*-values <0.03 by unpaired *t*-test) (Supplementary Figure S4A and D). Carfilzomib treatment increased A3A activity at all concentrations with fold changes ranging from 4.9 to 6.6 (Supplementary Figure S4B and E; all *P*-values <0.009 by unpaired *t*-test). Significant elevations in A3A activity were seen at both 90 and 270 nM ixazomib with a slightly higher fold change of 6.5 observed at 90 nM (Supplementary Figure S4C and F; *P*-values <0.005 by unpaired *t*-test).

Quantification of A3A transcript levels for all three drugs revealed similar trends. In BT474 cells, 13 and 65 nM bortezomib increased A3A 47.7- and 15.8-fold, respectively (Figure 6G), while 25, 50 and 100 nM carfilzomib increased A3A 31.2-, 21.9- and 16-fold in BT474 cells (Figure 6H). Finally, 90 and 270 nM ixazomib increased A3A 35.8- and 24.7-fold, respectively (Figure 6I), indicating that as with MG132, clinical proteasome inhibitors increase A3A abundance primarily through higher A3A mRNA abundance. Similar results were observed for bortezomib-, carfilzomib- and ixazomib-treated MDA-MB-453 cells (Supplementary Figure S4G–I).

### Proteasome inhibitors decrease cell growth and increase A3A-dependent DNA damage

**Figure 7.**
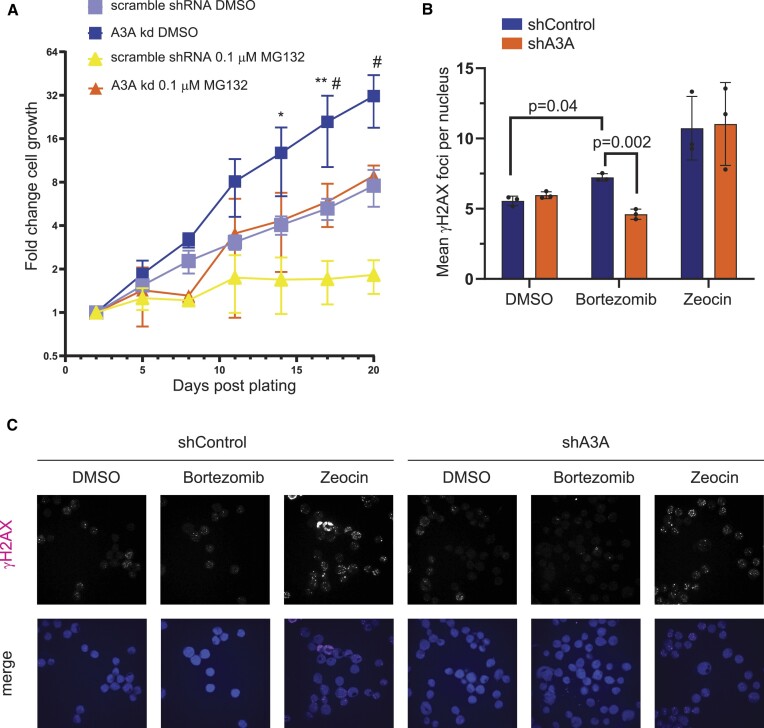
Proteasome inhibition leads to reduced cell growth. (**A**) Cell growth of MDA-MB-453 cells expressing an A3A-specific shRNA or scramble shRNA control treated with MG132 or DMSO over the span of 3 weeks counted by bright-field imaging. Plotted mean fold change in cell numbers at various days post-plating among replicates (*n* = 3). Error bars show one standard deviation. A3A shRNA expressing DMSO-treated cells, scramble shRNA expressing DMSO-treated cells, 0.1 μM MG132-treated A3A shRNA expressing cells, and 0.1 μM MG132-treated scramble shRNA cells are shown. Comparisons for each time point were made by multiple unpaired *t*-tests between scramble shRNA cells treated with DMSO and scramble shRNA cells treated with MG132 (statistical significance shown by an asterisk), as well as between MG132-treated scramble shRNA cells and A3A shRNA cells (statistical significance shown by a hash symbol). *^,#^*P* ≤ 0.05 and **P ≤ 0.01. All other time point comparisons not shown were not significant (*P* > 0.05). (**B**) The number of γH2AX foci per nuclei in MDA-MB-453 cells transduced to express either control shRNA (shControl) or A3A-targeting shRNA (shA3A). Statistical significance was determined by paired *t*-test. Error bars indicate standard deviation. (**C**) Representative γH2AX images merged with a Hoechst stain used for analysis plotted in Figure 6B.

To investigate how increased cellular A3A levels caused by proteasome inhibition affect breast cancer cells, we propagated MDA-MB-453 cells expressing either an shRNA scramble control or shRNA A3A knockdown in the absence or presence of 0.1 μM MG132 (Figure 7A). Cell growth was measured every 3 days over a span of 3 weeks with significant differences in fold change of observed cell growth emerging at 14 days post-plating between scramble shRNA cells treated with DMSO and scramble shRNA cells treated with MG132. Significant differences in cell growth between scramble-targeting shRNA and A3A-targeting shRNA knockdown lines treated with MG132 were apparent at 17 days post-plating, where MG132 treatment resulted in stagnant cell proliferation of MDA-MB-453 scramble shRNA expressing cells. In contrast, cells expressing an A3A-targeting shRNA continued to proliferate in the presence of MG132, although at a slower rate than either scramble- or A3A-targeting shRNA expressing cells treated with DMSO. Thus, A3A contributes to MG132 limiting cellular proliferation in MDA-MB-453 cells, possibly by increasing genomic DNA damage.

Because high levels of A3A can induce DNA double-strand breaks (DSBs) (55,56), we next tested whether proteasome inhibition elevates DSB formation in an A3A-dependent manner. We treated MDA-MB-453 cells expressing scramble control or A3A-targeting shRNA with either DMSO or bortezomib and used immunofluorescence to assess γH2AX as a marker of DSB formation (Figure 7B). Bortezomib-treated parental and scramble shRNA expressing MDA-MB-453 cells displayed higher numbers of γH2AX foci per nuclei compared to DMSO-treated controls. However, γH2AX foci were significantly diminished in A3A knockdown cells, indicating that the increase in A3A protein levels produced by proteasome inhibition results in increased DNA damage. While the increase in A3A-dependent γH2AX caused by bortezomib is relatively small, DSBs are a small subset of the DNA damage that A3A induces. Therefore, we expect the increased DSBs to represent a significant increase in replication-stalling abasic sites caused by A3A and UNG2 activity on the lagging strand template.

We also found that the ability of proteasome inhibition to increase A3A-dependent DSBs is cell line dependent. Similar analysis of γH2AX foci in BT474 cells expressing either control or A3A-targeting shRNAs revealed that bortezomib uniformly reduced DSB formation (Supplementary Figure S5). Moreover, the A3A-targeting shRNA had no impact on γH2AX foci in any treatment condition in BT474, suggesting that A3A-mediated production of DSBs may be limited in this cell line.

### Proteasome inhibition upregulates A3A in multiple myeloma cells

**Figure 8.**
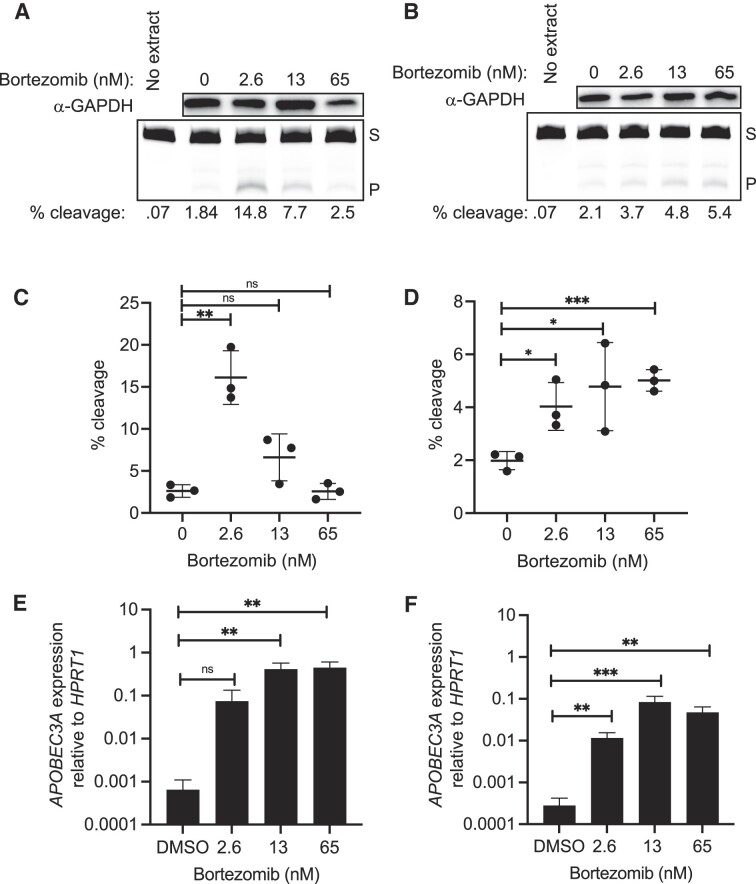
Bortezomib increases A3A in multiple myeloma cell lines. A3A abundance and activity after 24 h treatment with various concentrations of bortezomib in multiple myeloma cell lines MM.1S (**A**, **C**, **E**) and U266 (**B**, **D**, **F**). Representative images of GAPDH western blot analysis and denaturing gel of activity assays showing substrate cleavage in MM.1S cells (A) and U266 cells (B). (C, D) A3A activity was determined with the *in vitro* cytidine deaminase assay and substrate percent cleavage was plotted (*n* = 3). Shown is the mean of replicates and error bars that represent one standard deviation. Significant changes in activity for each line after bortezomib treatment are shown by asterisks, as determined by unpaired *t*-tests. (E) The transcript levels for A3A in MM.1S cells when compared to *HPRT1* increased significantly by 115-fold (ratio paired *t*-test; *P*-value 0.0801), 641-fold (ratio paired *t*-test; *P*-value 0.0052) and 688-fold (ratio paired *t*-test; *P*-value 0.0054) for 2.6, 13 and 65 nM bortezomib, respectively. (F) The transcript levels for A3A in U266 cells, when compared to *HPRT1*, increased significantly by 41.5-fold (ratio paired *t*-test; *P*-value 0.0052), 300-fold (ratio paired *t*-test; *P*-value 0.0003) and 171-fold (ratio paired *t*-test; *P*-value 0.0014) at 2.6, 13 and 65 nM, respectively. S, substrate; P, product. *P ≤ 0.05, **P ≤ 0.01 and ***P ≤ 0.001; ns, non-significant with *P*-value >0.05.

Development of bortezomib as a first-generation proteasome inhibitor anticancer drug has been critical to the successful treatment of multiple myeloma patients; however, relapse does occur. Because of the clinical use of bortezomib and the interest to develop new-generation proteasome inhibitors for multiple myeloma treatment, we wanted to characterize the relationship these drugs have with A3A abundance and activity in multiple myeloma cells. We performed the same experiments as described earlier with breast cancer cell lines, on two multiple myeloma cell lines, MM.1S and U266. After a 24 h treatment with bortezomib at various concentrations, we measured the transcript and activity levels for A3A (Figure 8). In MM.1S cells, A3A activity on the ssDNA substrate peaked at 2.6 nM bortezomib with a fold change of 6.2 in substrate cleavage compared to DMSO (*P* = 0.002, unpaired *t*-test) (Figure 8A and C). The transcript levels for A3A in MM.1S cells increased significantly by 115-, 641- and 688-fold for 2.6, 13 and 65 nM, respectively (Figure 8E). In U266 cells, increasing concentrations of bortezomib treatment resulted in greater elevations in A3A activity and transcripts (Figure 8B, D and F). A maximum increase of 2.5-fold at 65 nM relative to DMSO was observed for A3A activity (all *P*-values <0.05, unpaired *t*-test) (Figure 8B and D). The fold change in observed transcript levels maxed out at ∼300-fold at 13 nM (Figure 8F).

## Discussion

Recently, A3A has been identified as a major source of cytidine deaminase-induced mutations in breast cancer (16–18,57), which has emphasized the importance of understanding what mechanisms influence A3A abundance in cancer cells. We show here that dysregulation of the ubiquitin-proteasome system, either by blocking proteasome function with drugs or by reducing expression of specific proteins in ubiquitin ligase complexes, increases A3A in a variety of cancer cell types. In breast cancer cells, proteasome inhibition can result in an A3A-dependent reduction of cell proliferation and increased DNA damage, suggesting that clinical utilization of these drugs in cancer treatment may induce genetic heterogeneity within the tumor.

Surprisingly, increased A3A abundance in cancer cell lines appears to primarily result from elevated *APOBEC3A* transcription instead of blocking proteasome-mediated A3A destruction. We hypothesize that the proteasome is responsible for degrading a critical transcription factor that regulates A3A transcription. By blocking proteasome activity with a drug, the amount of the transcription factor increases and in turn increases A3A transcript levels. In line with this theory, knockdown of FBXO22, an F-Box protein functioning in E3 ligase complexes, increases A3A transcript levels. F-box proteins have emerged as important components of the ubiquitin-proteasome system that are critical for recognition and regulation of many cell cycle regulatory proteins and transcription factors by ubiquitin ligase complexes (49). This suggests that FBXO22 mediates the destruction of a transcription factor for A3A, and that blocking the ubiquitin-proteasome system with inhibitors has a similar effect of increasing the presence of a transcription factor that is upregulating A3A transcription. Alternatively, proteasome inhibition could initiate cell stress responses whose signaling activates transcription factors that happen to also regulate A3A transcription. In this context, proteasome inhibition would indirectly elevate A3A as part of a transcriptional program responding to loss of either proteasome or FBXO22 activity.

In either case, the identity of the transcription factor regulating A3A expression upon proteasome inhibition remains unknown. Rel-A and STAT2, which both increase A3A expression in response to DNA damage or innate immune signaling, respectively, are not required for the increase of A3A transcript upon proteasome dysfunction, indicating that it results from a novel form of transcriptional regulation. Based upon proximity of known transcription factor binding sites to the start sites of nascent A3A mRNA following MG132 treatment (as measured by csRNA-seq), an Ets or bZIP family transcription factor seems most likely. This is additionally supported by bZIP and Ets factors being the second and fifth most enriched transcription factor binding sites occurring in promoters of genes induced >2-fold by MG132 treatment (Supplementary Figure S2). However, the high redundancy of these families of transcription factors makes identification of the specific factor driving A3A transcription in response to proteasome inhibition difficult (58). We also note that transcripts from both the endogenous A3A locus and exogenously expressed A3A cDNA are elevated upon proteasome inhibition. The promoter regions of these two genes are vastly different, suggesting that A3A mRNA levels may be controlled either by elements within the A3A coding sequence or by common distal enhancers flanking the A3A locus and the site of A3A cDNA integration following lentiviral transduction.

The mechanism of A3A transcriptional regulation being altered by proteasome inhibitors appears to be conserved across cell types. We showed that A3A can be exogenously overexpressed in immortalized retinal pigment epithelial cells and still have a significant increase in transcript levels when the ubiquitin-proteasome system is inhibited. A similar relationship occurs in breast cancer as well as multiple myeloma cells. However, each cell line tested displayed vastly different fold increases in A3A mRNA during proteasome inhibition. These differences could stem from differences in proteasome subunit activity between specific cell types, as the proteasome inhibitors tested have differing activity on proteasomal subunits. Alternatively, differences in the basal A3A expression level among cell lines could result in higher fold changes among cells with low A3A expression prior to proteasome inhibitor treatment.

Increases in A3A cytidine deaminase activity generally corresponded with changes in A3A transcript levels across the retinal pigment epithelial, breast cancer and multiple myeloma cell lines tested. However, in MM.1S cells, following an initial increase, A3A activity levels decreased while transcript levels remained elevated. This difference between A3A activity and transcript level could result from proteasome impacts on an unknown cell type-specific mechanism of A3A inhibition. Proteasome inhibitors produce a wide range of cellular impacts such as endoplasmic reticulum and mitochondrial stress, p53 activation, decreased growth factor receptors and reactive oxygen species production (59). Thus, proteasome inhibition could result in activation of an A3A inhibitor either indirectly through stress response signaling pathways or directly by preventing the degradation of potential A3A post-translational modifying enzymes or A3A interacting proteins that could reduce A3A catalytic activity.

Altered functionality of the ubiquitin-proteasome system through MG132 treatment or activity of ARIH1-, UBE2L3- and pVHL-containing ubiquitin ligase complexes also increases A3A protein levels in human embryonic kidney cells and hepatocytes (22,23). However, these impacts were attributed to direct proteasome degradation of A3A instead of altered transcript levels. The extent to which direct proteasome-mediated degradation of A3A influences the protein’s cellular abundance remains to be determined. While ubiquitinated A3A has been reportedly detected, whether this occurs to a sufficient level to alter steady-state endogenous A3A protein levels is unknown as are the cell type specificity of A3A degradation, potential sites of A3A ubiquitination and the mechanisms potentially inducing its degradation.

Our results of increased A3A abundance and DNA damage add important considerations to what factors may be impacting patient responses to proteasome inhibitors. Bortezomib, carfilzomib and ixazomib are used in standard care treatments for mantle cell lymphoma and multiple myeloma. As 30% of tumors are APOBEC mutated (12), the effects of proteasome inhibitors on A3A and the consequences of elevated cellular A3A have the potential to affect a great number of patients currently receiving proteasome inhibitor therapy. However, it is unclear exactly what significance increased A3A protein and activity may have in patient responses to proteasome inhibitors and the efficacy of other standard care therapies. We consider two potential roles here. Increased nuclear DNA damage induced by increased A3A abundance in cancer cells of patients receiving proteasome inhibitors leads to mutations that could lead to greater susceptibility of the cancer cells to targeted therapies and improved patient responses with an increased efficacy of targeted treatments. This idea is supported by other studies that have found APOBEC mutagenesis benefits for therapy responses in patients with other cancers (60,61). Targeted therapies that could have increased efficacy include DNA damage targeting therapies or immunotherapies against neoantigens as increased tumor mutation load may lead to novel immunogenic cell surface peptides. Alternatively, an induced increase in DNA damage in cancer cells could lead to higher genetic heterogeneity within a tumor population and provide a means for future therapeutic resistance and worse patient outcomes, as supported by (62) in non-small cell lung cancer, and in (63) where APOBEC mutations significantly correlated with a worse prognosis in the highest APOBEC mutated multiple myeloma samples.

## Supplementary Material

zcad058_Supplemental_Files

## Data Availability

This manuscript utilizes qRT-PCR to evaluate gene expression as well as newly synthesized oligonucleotides for amplification and shRNA expression. Details of qRT-PCR conditions are provided in the ‘Materials and methods’ section. Oligonucleotide sequences are provided in Supplementary Table S1. Raw Cq values are provided in Supplementary Table S2. csRNA-seq data have been deposited at the GEO (https://www.ncbi.nlm.nih.gov/geo/) under accession number GSE248016. Log_2_ fold change values for all transcripts measured by csRNA-seq are provided in Supplementary Table S3.
