# Supplementary material for: An impaired ubiquitin-proteasome system increases APOBEC3A abundance
Source: NAR Cancer. 2023 Dec 19;5(4):zcad058. doi: 10.1093/narcan/zcad058 (PMC10753533; doi:10.1093/narcan/zcad058)
Supplement: zcad058_Supplemental_Files [file zcad058_supplemental_files.zip › Supplemental Information_v3_clean.pdf]

Supplemental Information

Sequence of pTM-682

LOCUS   pTM-682   9443 bp   DNA   circular UNA 08-NOV-2023
DEFINITION an expression clone created from pTM-633 (reversed) and pTM 190
            pENTR with A3A with 3X HA tag.
ACCESSION   urn.local...i-gz1t54w
VERSION   urn.local...i-gz1t54w
KEYWORDS   .
SOURCE
  ORGANISM .
FEATURES   Location/Qualifiers
     rep_origin   complement(3..458)
     misc_feature   complement(843..964)
                     /note="Geneious type: polyA_signal"
                     /note="SV40 poly(A) signal"
                     /note="SV40 polyadenylation signal"
                     /standard_name="SV40 poly(A) signal polyA signal"
     repeat_region   1036..1269
                     /note="3′ long terminal repeat (LTR) from HIV-1
                     (self-inactivating)"
                     /note="Derived using Geneious Prime 2023.2.1 'Annotate
                     from Database' based on nucleotide similarity"
                     /standard_name="3′ LTR (ΔU3)"
                     /note="Geneious type: LTR"
     CDS   complement(1360..2163)
                     /codon_start=1
                     /gene="aph(3')-II (or nptII)"
                     /product="aminoglycoside phosphotransferase from Tn5"
                     /note="NeoR/KanR"
                     /note="confers resistance to neomycin, kanamycin, and
                     G418(Geneticin(R))"
                     /translation="MGSAIEQDGLHAGSPAAWVERLFGYDWAQQTIGCSDAAVFRLSA
                     QGRPVLFVKTDLSGALNELQDEAARLSWLATTGVPCAAVLDVVTEAGRDWLLLGEVPG
                     QDLLSSHLAPAEKVSIMADAMRRLHTLDPATCPFDHQAKHRIERARTRMEAGLVDQDD
                     LDEEHQGLAPAELFARLKARMPDGEDLVVTHGDACLPNIMVENGRFSGFIDCGRLGVA
                     DRYQDIALATRDIAEELGGEWADRFLVLYGIAAPDSQRIAFYRLLDEFF"
                     /standard_name="NEO_G418R"
     regulatory   complement(2177..2676)
                     /note="Geneious type: promoter"
                     /note="Mus musculus phosphoglycerate kinase 1 promoter"
                     /regulatory_class="promoter"
                     /note="Derived using Geneious Prime 2023.2.1 'Annotate

```
             from Database' based on nucleotide similarity"
             /standard_name="PGK"
regulatory      2707..3335
             /note="Geneious type: promoter"
             /standard_name="CMV-weak many base changes"
misc_feature    3216..3255
             /gene="2X TetO2"
             /standard_name="2X TetO2"
misc_feature    3374..3394
             /note="Geneious type: attB1"
             /standard_name="attB1"
CDS             join(3421..3889,4306..4580)
             /standard_name="A3A CDS"
intron          3890..4305
             /standard_name="INTRON"
misc_feature    4440..4469
             /standard_name="FLEXIBlE linker"
misc_feature    4470..4577
             /standard_name="3X HA tag"
misc_feature    4578..4580
             /standard_name="STOP codon"
misc_feature    complement(4623..4643)
             /note="Geneious type: attB2"
             /standard_name="attB2"
misc_feature    4675..5263
             /note="WPRE"
             /note="woodchuck hepatitis virus posttranscriptional
             regulatory element"
             /standard_name="WPRE"
misc_feature    complement(5315..5432)
             /note="cPPT/CTS"
             /note="central polypurine tract and central termination
             sequence of HIV-1"
             /standard_name="cPPT/CTS"
misc_feature    complement(5921..6154)
             /note="RRE"
             /note="The Rev response element (RRE) of HIV-1 allows for
             Rev-dependent mRNA export from the nucleus to
             thecytoplasm."
             /standard_name="RRE"
misc_feature    complement(6816..6996)
             /note="Geneious type: LTR"
             /note="5' LTR (truncated)"
             /note="truncated 5' long terminal repeat (LTR) from HIV-1"
```

/standard_name="5' LTR (truncated)"
rep_origin complement(7693..8281)
CDS complement(8452..9312)
/codon_start=1
/gene="bla"
/product="beta-lactamase"
/note="AmpR"
/note="confers resistance to ampicillin, carbenicillin,
and related antibiotics"
/translation="MSIQHFRVALIPFFAAFCLPVFAHPETLVKVKDAEDQLGARVGY
IELDLNSGKILESFRPEERFPMMSTFKVLLCGAVLSRIDAGQEQLGRRIHYSQNDLVE
YSPVTEKHLTDGMTVRELCSAAITMSDNTAANLLLTTIGGPKELTAFLHNMGDHVTRL
DRWEPELNEAIPNDERDTTMPVAMATTLRKLLTGELLTLASRQQLIDWMEADKVAGPL
LRSALPAGWFIADKSGAGERGSRGIIAALGPDGKPSRIVVIYTTGSQATMDERNRQIA
EIGASLIKHW"
/standard_name="bla CDS"
regulatory complement(9313..9417)
/note="Geneious type: promoter"
/gene="bla"
/note="AmpR promoter"
/standard_name="bla promoter"

ORIGIN

```
        1 ggaaattgta aacgttaata ttttgttaaa attcgcgtta aatttttgtt aaatcagctc
       61 attttttaac caataggccg aaatcggcaa aatcccttat aaatcaaaag aatagaccga
      121 gatagggttg agtgttgttc cagtttggaa caagagtcca ctattaaaga acgtggactc
      181 caacgtcaaa gggcgaaaaa ccgtctatca gggcgatggc ccactacgtg aaccatcacc
      241 ctaatcaagt tttttggggt cgaggtgccg taaagcacta aatcggaacc ctaaagggag
      301 cccccgattt agagcttgac ggggaaagcc ggcgaacgtg gcgagaaagg aagggaagaa
      361 agcgaaagga gcgggcgcta gggcgctggc aagtgtagcg gtcacgctgc gcgtaaccac
      421 cacacccgcc gcgcttaatg cgccgctaca gggcgcgtcg cgccattcgc cattcaggct
      481 gcgcaactgt tgggaagggc gatcggtgcg ggcctcttcg ctattacgcc agctggcgaa
      541 agggggatgt gctgcaaggc gattaagttg ggtaacgcca gggttttccc agtcacgacg
      601 ttgtaaaacg acggccagtg agcgcgcgta atacgactca ctatagggcg aattgggtac
      661 gtccctaggc ctccaaaaaa gcctcctcac tacttctgga atagctcaga ggccgaggcg
      721 gcctcggcct ctgcataaat aaaaaaaatt agtcagccat ggggcggaga atgggcggaa
      781 ctgggcggag ttaggggcgg gatgggcgga gttaggggcg ggatagctag agccagacat
      841 gataagatac attgatgagt ttggacaaac cacaactaga atgcagtgaa aaaaatgctt
      901 tatttgtgaa atttgtgatg ctattgcttt atttgtaacc attataagct gcaataaaca
      961 agttcctctc actctctgat attcatttct ttgcaagtta taaatactga ataataagat
     1021 gacatgaact actactgcta gagattttcc acactgacta aaagggtctg agggatctct
     1081 agttaccaga gtcacacaac agacgggcac acactacttg aagcactcaa ggcaagcttt
     1141 attgaggctt aagcagtggg ttccctagtt agccagagag ctcccaggct cagatctggt
     1201 ctaaccagag agacccagta caagcaaaaa gcagatcttg tcttcgttgg gagtgagcta
     1261 gcccttccag tccccccttt tcttttaaaa agtggctaag atctacagct gccttgtaag
     1321 tcattggtct taaaggtacc gggccctcga cggatcccct cagaagaact cgtcaagaag
     1381 gcgatagaag gcgatgcgct gcgaatcggg agcggcgata ccgtaaagca cgaggaagcg
     1441 gtcagcccat tcgccgccaa gctcttcagc aatatcacgg gtagccaacg ctatgtcctg
     1501 atagcggtcc gccacaccca gccggccaca gtcgatgaat ccagaaaagc ggccattttc
     1561 caccatgata ttcggcaagc aggcatcgcc atgggtcacg acgagatcct cgccgtcggg
     1621 catgcgcgcc ttgagcctgg cgaacagttc ggctggcgcg agcccctgat gctcttcgtc
```

1681 cagatcatcc tgatcgacaa gaccggcttc catccgagta cgtgctcgct cgatgcgatg
1741 tttcgcttgg tggtcgaatg ggcaggtagc cggatcaagc gtatgcagcc gccgcattgc
1801 atcagccatg atggatactt tctcggcagg agcaaggtga gatgacagga gatcctgccc
1861 cggcacttcg cccaatagca gccagtccct tcccgcttca gtgacaacgt cgagcacagc
1921 tgcgcaagga acgcccgtcg tggccagcca cgatagccgc gctgcctcgt cctgcagttc
1981 attcagggca ccggacaggt cggtcttgac aaaaagaacc gggcgcccct gcgctgacag
2041 ccggaacacg gcggcatcag agcagccgat tgtctgttgt gcccagtcat agccgaatag
2101 cctctccacc caagcggccg gagaacctgc gtgcaatcca tcttgttcaa tggccgatcc
2161 catattggct gcaggtcgaa aggcccggag atgaggaaga ggagaacagc gcggcagacg
2221 tgcgcttttg aagcgtgcag aatgccgggc ctccggagga ccttcgggcg cccgccccgc
2281 ccctgagccc gcccctgagc ccgcccccgg acccacccct tcccagcctc tgagcccaga
2341 aagcgaagga gcaaagctgc tattggccgc tgccccaaag gcctacccgc ttccattgct
2401 cagcggtgct gtccatctgc acgagactag tgagacgtgc tacttccatt tgtcacgtcc
2461 tgcacgacgc gagctgcggg gcggggggga acttcctgac taggggagga gtagaaggtg
2521 gcgcgaaggg gccaccaaag aacggagccg gttggcgcct accggtggat gtggaatgtg
2581 tgcgaggcca gaggccactt gtgtagcgcc aagtgcccag cggggctgct aaagcgcatg
2641 ctccagactg ccttgggaaa agcgcctccc ctacccggta gaattagatc actagtccgg
2701 ataacgcgtt acataactta cggtaaatgg cccgcctggc tgaccgccca acgacccccg
2761 cccattgacg tcaataatga cgtatgttcc catagtaacg ccaataggga ctttccattg
2821 acgtcaatgg gtggagtatt tacggtaaac tgcccacttg gcagtacatc aagtgtatca
2881 tatgccaagt acgcccccta ttgacgtcaa tgacggtaaa tggcccgcct ggcattatgc
2941 ccagtacatg accttatggg actttcctac ttggcagtac atctacgtat tagtcatcgc
3001 tattaccatg gtgatgcggt tttggcagta catcaatggg cgtggatagc ggtttgactc
3061 acggggattt ccaagtctcc accccattga cgtcaatggg agtttgtttt ggcaccaaaa
3121 tcaacgggac tttccaaaat gtcgtaacaa ctccgcccca ttgacgcaca tggccggaag
3181 gcgtgaactg tgagaggtct atataagcag agctctccct atcagtgata gagatctccc
3241 tatcagtgat agagatcgtc gacgagctcg ttttgagaac cctcatatcg cctggagacg
3301 ccatccacgc tgttttgacc tccatagaag acaccgactc tagtccagtg tggtggaatt
3361 ctgcagatat caacaagttt gtacaaaaaa gcaggcttta aggaaccaa ttcagtcatc
3421 atggaagcca gcccagcatc cggcccaga cacttgatgg atccacacat attcacttcc
3481 aactttaaca atggcattgg aaggcataag acctacctgt gctacgaagt ggagcgcctg
3541 gacaatggca cctcggtcaa gatggaccag cacaggggct ttctacacaa ccaggctaag
3601 aatcttctct gtggctttta cggccgccat gcggagctgc gcttcttgga cctggttcct
3661 tctttgcagt tggacccggc ccagatctac agggtcactt ggttcatctc ctggagcccc
3721 tgcttctcct ggggctgtgc cggggaagtg cgtgcgttcc ttcaggagaa cacacacgtg
3781 agactgcgta tcttcgctgc ccgcatctat gattacgacc ccctatataa ggaggcactg
3841 caaatgctgc gggatgctgg ggcccaagtc tccatcatga cctacgatgg taagaatgga
3901 aggttcaggt ggggtggggt gggtgggggc aggagaggtt cctgggaaga aaaggagaaa
3961 ggccttggtc tgctgcctgc agaaacgatg gctggactct gggacctgac tttggggtcg
4021 atgggaagag agaggccagg ccaggagatg tgggcccagg gagggcaggg agagtggctg
4081 gaagtggaag cagaacttgg ggctttctga aagaatgaga actgggctgg cccagattcc
4141 aatgggaagg aactgcctga tgaaggagct aagtccctag ggagggaga gggaaaggag
4201 ggactgaaac caggatgtgg gaagtctgtc ctgagagtca tgggccctag gtgccacccc
4261 gatcccacag cgggagcgtg acttatctcc cctgtccctt ttcagaattt aagcactgct
4321 gggacacctt tgtggaccac cagggatgtc ccttccagcc ctgggatgga ctagatgagc
4381 acagccaagc cctgagtggg aggctgcggg ccattctcca gaatcaggga aacaccggtg
4441 gaggaggcgg atccggagga ggcggatccg cggccgttta cccatacgat gttcctgact
4501 atgcgggcta tccctatgac gtcccggact atgcaggatc ctatccatat gacgttccag
4561 attacgctcc ggccgcctag accggttagg gtacccacag gagaacgggg tgtatctaga
4621 cccagctttc ttgtacaaag tggttgatat ccagcacagt ggcggccgct cgacaatcaa
4681 cctctggatt acaaaatttg tgaaagattg actggtattc ttaactatgt tgctcctttt
4741 acgctatgtg gatacgctgc tttaatgcct ttgtatcatg ctattgcttc ccgtatggct
4801 ttcattttct cctccttgta taaatcctgg ttgctgtctc tttatgagga gttgtggccc
4861 gttgtcaggc aacgtggcgt ggtgtgcact gtgtttgctg acgcaacccc cactggttgg
4921 ggcattgcca ccacctgtca gctcctttcc gggactttcg ctttcccct ccctattgcc
4981 acggcggaac tcatcgccgc ctgccttgcc cgctgctgga caggggctcg gctgttgggc
5041 actgacaatt ccgtggtgtt gtcggggaag ctgacgtcct ttccatggct gctcgcctgt

5101 gttgccacct ggattctgcg cgggacgtcc ttctgctacg tcccttcggc cctcaatcca
5161 gcggaccttc cttcccgcgg cctgctgccg gctctgcggc ctcttccgcg tcttcgcctt
5221 cgccctcaga cgagtcggat ctccctttgg gccgcctccc cgcctggaat tctgcagata
5281 tccggactag tgatctaatt ctaccgttat cgataaaatt ttgaattttt gtaatttgtt
5341 tttgtaattc tttagtttgt atgtctgttg ctattatgtc tactattctt tcccctgcac
5401 tgtacccccc aatcccccct tttcttttaa aagttaaccg tcgagatccg ttcactaatc
5461 gaatggatct gtctctgtct ctctctccac cttcttcttc tattccttcg ggcctgtcgg
5521 gtcccctcgg ggttgggagg tgggtctgaa acgataatgg tgaatatccc tgcctaactc
5581 tattcactat agaaagtaca gcaaaaacta ttcttaaacc taccaagcct cctactatca
5641 ttatgaataa ttttatatac cacagccaat ttgttatgtt aaaccaattc cacaaacttg
5701 cccatttatc taattccaat aattcttgtt cattcttttc ttgctggttt tgcgattctt
5761 caattaagga gtgtattaag cttgtgtaat tgttaatttc tctgtcccac tccatccagg
5821 tcgtgtgatt ccaatctgtt ccagagattt attactccaa ctagcattcc aaggcacagc
5881 agtggtgcaa atgagttttc cagagcaacc ccaaatcccc aggagctgtt gatcctttag
5941 gtatctttcc acagccagga ttcttgcctg gagctgcttg atgccccaga ctgtgagttg
6001 caacagatgc tgttgcgcct caatagccct cagcaaattg ttctgctgct gcactatacc
6061 agacaataat tgtctggcct gtaccgtcag cgtcattgag gctgcgccca tagtgcttcc
6121 tgctgctccc aagaacccaa ggaacaaagc tcctattccc actgctcttt tttctctctg
6181 caccactctt ctctttgcct tggtgggtgc tactcctaat ggttcaattt ttactacttt
6241 atatttatat aattcacttc tccaattgtc cctcatatct cctcctccag gtctgaagat
6301 cagcggccgc ttgctgtgcg gtggtcttac ttttgttttg ctcttcctct atcttgtcta
6361 aagcttcctt ggtgtctttt atctctatcc tttgatgcac acaatagagg gttgctactg
6421 tattatataa tgatctaagt tcttctgatc ctgtctgaag ggatggttgt agctgtccca
6481 gtatttgtct acagccttct gatgtttcta acaggccagg attaactgcg aatcgttcta
6541 gctccctgct tgcccatact atatgtttta atttatattt tttctttccc cctggcctta
6601 accgaatttt ttcccatcgc gatctaattc tcccccgctt aatactgacg ctctcgcacc
6661 catctctctc cttctagcct ccgctagtca aaatttttgg cgtactcacc agtcgccgcc
6721 cctcgcctct tgccgtgcgc gcttcagcaa gccgagtcct gcgtcgagag agctctggtt
6781 tccctttcgc tttcaggtcc ctgttcgggc gccactgcta gagattttcc acactgacta
6841 aaagggtctg agggatctct agttaccaga gtcacacaac agacgggcac acactacttg
6901 aagcactcaa ggcaagcttt attgaggctt aagcagtggg ttccctagtt agccagagag
6961 ctcccaggct cagatctggt ctaaccagag agacccgttt attgtatcga gctaggcact
7021 taaatacaat atctctgcaa tgcggcaatt cagtggttcg tccaatccat gtcagacccg
7081 tctgttgcct tcctaataag gcacgatcgt accaccttac ttccaccaat cggcatgcac
7141 ggtgcttttt ctctccttgt aaggcatgtt gctaactcat cgttaccatg ttgcaagact
7201 acaagagtat tgcataagac tacattaagc ttgcagctcc agcttttgtt ccctttagtg
7261 agggttaatt gcgcgcttgg cgtaatcatg gtcatagctg tttcctgtgt gaaattgtta
7321 tccgctcaca attccacaca acatacgagc cggaagcata aagtgtaaag cctggggtgc
7381 ctaatgagtg agctaactca cattaattgc gttgcgctca ctgcccgctt tccagtcggg
7441 aaacctgtcg tgccagctgc attaatgaat cggccaacgc gcggggagag gcggtttgcg
7501 tattgggcgc tcttccgctt cctcgctcac tgactcgctg cgctcggtcg ttcggctgcg
7561 gcgagcggta tcagctcact caaaggcggt aatacggtta tccacagaat caggggataa
7621 cgcaggaaag aacatgtgag caaaaggcca gcaaaaggcc aggaaccgta aaaaggccgc
7681 gttgctggcg tttttccata ggctccgccc ccctgacgag catcacaaaa atcgacgctc
7741 aagtcagagg tggcgaaacc cgacaggact ataaagatac caggcgtttc cccctggaag
7801 ctccctcgtg cgctctcctg ttccgaccct gccgcttacc ggatacctgt ccgcctttct
7861 cccttcggga agcgtggcgc tttctcatag ctcacgctgt aggtatctca gttcggtgta
7921 ggtcgttcgc tccaagctgg gctgtgtgca cgaacccccc gttcagcccg accgctgcgc
7981 cttatccggt aactatcgtc ttgagtccaa cccggtaaga cacgacttat cgccactggc
8041 agcagccact ggtaacagga ttagcagagc gaggtatgta ggcggtgcta cagagttctt
8101 gaagtggtgg cctaactacg gctacactag aaggacagta tttggtatct gcgctctgct
8161 gaagccagtt accttcggaa aaagagttgg tagctcttga tccggcaaac aaaccaccgc
8221 tggtagcggt ggtttttttg tttgcaagca gcagattacg cgcagaaaaa aaggatctca
8281 agaagatcct ttgatctttt ctacggggtc tgacgctcag tggaacgaaa actcacgtta
8341 agggattttg gtcatgagat tatcaaaaag gatcttcacc tagatccttt taaattaaaa
8401 atgaagtttt aaatcaatct aaagtatata tgagtaaact tggtctgaca gttaccaatg
8461 cttaatcagt gaggcaccta tctcagcgat ctgtctattt cgttcatcca tagttgcctg

```
8521 actccccgtc gtgtagataa ctacgatacg ggagggctta ccatctggcc ccagtgctgc
8581 aatgataccg cgagacccac gctcaccggc tccagattta tcagcaataa accagccagc
8641 cggaagggcc gagcgcagaa gtggtcctgc aactttatcc gcctccatcc agtctattaa
8701 ttgttgccgg gaagctagag taagtagttc gccagttaat agtttgcgca acgttgttgc
8761 cattgctaca ggcatcgtgg tgtcacgctc gtcgtttggt atggcttcat tcagctccgg
8821 ttcccaacga tcaaggcgag ttacatgatc ccccatgttg tgcaaaaaag cggttagctc
8881 cttcggtcct ccgatcgttg tcagaagtaa gttggccgca gtgttatcac tcatggttat
8941 ggcagcactg cataattctc ttactgtcat gccatccgta agatgctttt ctgtgactgg
9001 tgagtactca accaagtcat tctgagaata gtgtatgcgg cgaccgagtt gctcttgccc
9061 ggcgtcaata cgggataata ccgcgccaca tagcagaact ttaaaagtgc tcatcattgg
9121 aaaacgttct tcggggcgaa aactctcaag gatcttaccg ctgttgagat ccagttcgat
9181 gtaacccact cgtgcaccca actgatcttc agcatctttt actttcacca gcgtttctgg
9241 gtgagcaaaa acaggaaggc aaaatgccgc aaaaaaggga ataagggcga cacggaaatg
9301 ttgaatactc atactcttcc tttttcaata ttattgaagc atttatcagg gttattgtct
9361 catgagcgga tacatatttg aatgtattta gaaaaataaa caaatagggg ttccgcgcac
9421 atttccccga aaagtgccac ctg
```
//

Supplemental Table 1: Sequences of shRNA targets and oligonucleotides

Supplemental Table 2: Raw Cq values for qRT-PCR

Supplemental Table 3: Log_2 csRNA-seq reads per million values for all transcripts sorted by greatest to least fold change.

Supplemental Figure 1

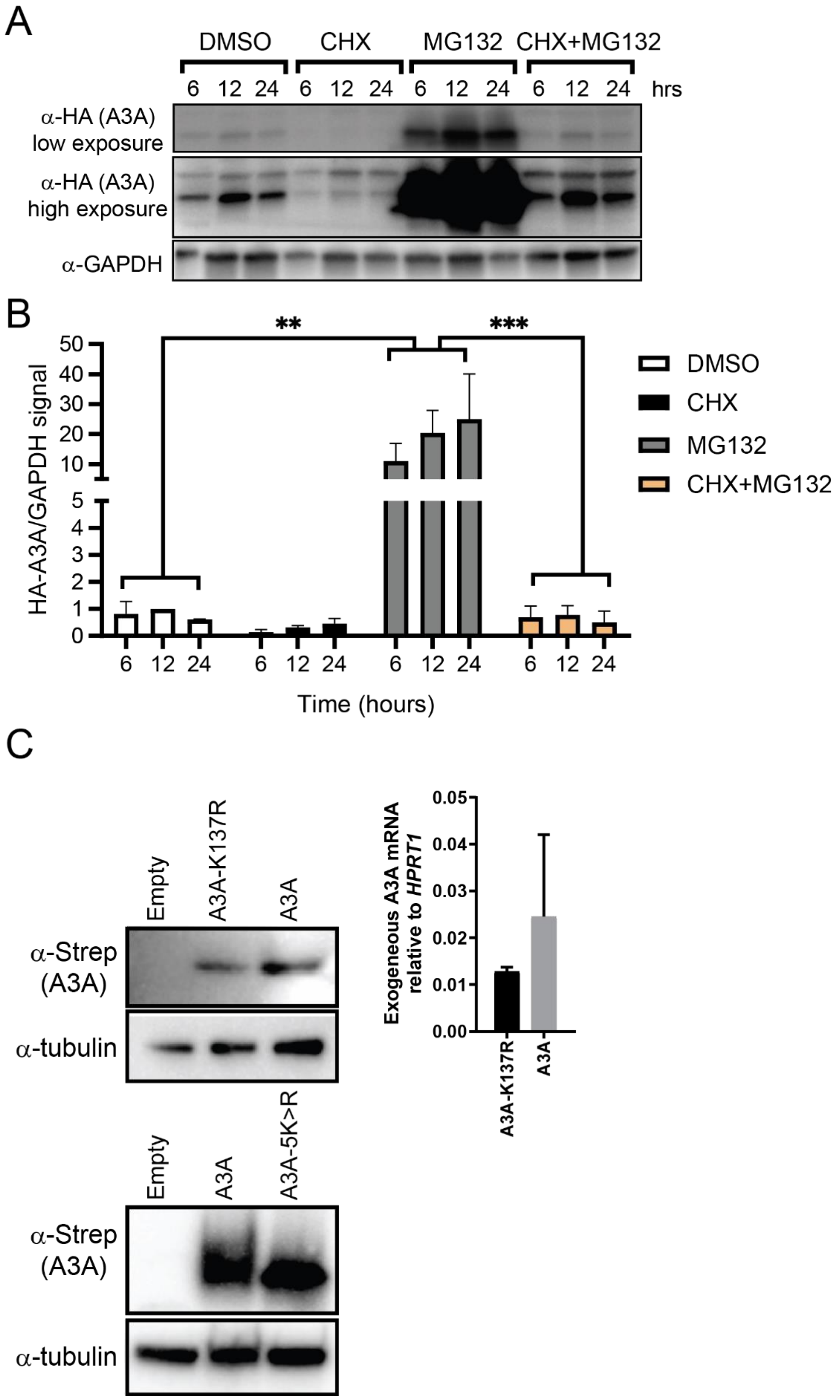

Supplemental Figure 1: APOBEC3A is primarily elevated during proteasome inhibition with MG132 by increased transcription. RPE-1 cells that had been transduced with HA-tagged A3A were treated with 300 μg/ml cycloheximide, 0.5 μM MG132, or both and samples collected at 6, 12, and 24 hours for n=3 replicates. (A) Western blot analysis allowed quantification of A3A at all timepoints via an anti-HA antibody and GAPDH was used to ensure equal loading. Representative images of a GAPDH loading control and HA-A3A western blot at low and high exposure are included for visualization of all sample bands. (B) Plotted relative HA-A3A to GAPDH band signal intensity grouped by treatment. Bars indicate mean values. Error bars = standard deviation for three replicates. ** = p value < 0.01, *** = p value < 0.001. (C) Representative western blots show the abundance of Strep-A3A, Strep-A3A K137R mutant, and Strep-A3A 5K>R mutant in RPE-1 cells. Exogeneous A3A expression for WT A3A and A3A K137R was determined by qRT-PCR. Bars indicate mean values. Error bars indicate standard deviation of 2 biological replicates.

Supplemental Figure 2

A

Rank	Motif	P-value	log P-pvalue	% of Targets	% of Background	STD(Bg STD)	Best Match/Details
1	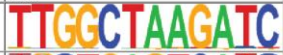	1e-109	-2.515e+02	3.26%	0.03%	34.3bp (79.8bp)	POL004.1_CCAAT-box/Jaspar(0.615) More Information | Similar Motifs Found
2	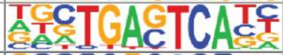	1e-99	-2.289e+02	13.87%	2.97%	56.4bp (75.1bp)	Jun-AP1(bZIP)/K562-cJun-ChIP-Seq(GSE31477)/Homer(0.977) More Information | Similar Motifs Found
3	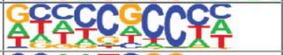	1e-89	-2.057e+02	31.98%	14.34%	50.2bp (75.9bp)	Sp2(Zf)/HEK293-Sp2.eGFP-ChIP-Seq(Encode)/Homer(0.892) More Information | Similar Motifs Found
4	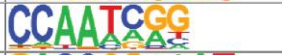	1e-81	-1.877e+02	21.22%	7.67%	62.5bp (79.9bp)	NFY(CCAAT)/Promoter/Homer(0.915) More Information | Similar Motifs Found
5	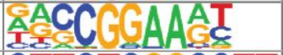	1e-72	-1.664e+02	21.92%	8.70%	57.8bp (82.6bp)	Elk1(ETS)/Hela-Elk1-ChIP-Seq(GSE31477)/Homer(0.974) More Information | Similar Motifs Found
6	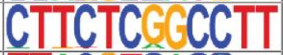	1e-52	-1.213e+02	1.73%	0.02%	1.0bp (77.1bp)	ZNF711(Zf)/SHSY5Y-ZNF711-ChIP-Seq(GSE20673)/Homer(0.617) More Information | Similar Motifs Found
7	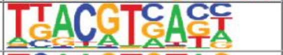	1e-50	-1.159e+02	21.62%	10.22%	57.5bp (79.4bp)	Atf1(bZIP)/K562-ATF1-ChIP-Seq(GSE31477)/Homer(0.920) More Information | Similar Motifs Found
8	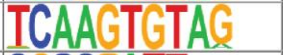	1e-45	-1.040e+02	2.02%	0.07%	49.2bp (74.0bp)	NKX2-8/MA0673.1/Jaspar(0.777) More Information | Similar Motifs Found
9	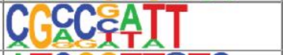	1e-40	-9.213e+01	29.91%	17.76%	69.4bp (84.2bp)	E2F2/MA0864.2/Jaspar(0.608) More Information | Similar Motifs Found
10	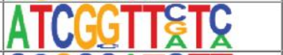	1e-35	-8.202e+01	1.38%	0.03%	40.3bp (56.9bp)	PH0044.1_Homez/Jaspar(0.703) More Information | Similar Motifs Found
11	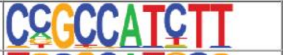	1e-30	-7.135e+01	6.91%	2.17%	67.1bp (73.4bp)	YY1(Zf)/Promoter/Homer(0.941) More Information | Similar Motifs Found
12	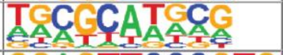	1e-28	-6.633e+01	10.12%	4.23%	59.5bp (83.9bp)	NRF(NRF)/Promoter/Homer(0.828) More Information | Similar Motifs Found
13	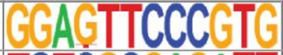	1e-22	-5.185e+01	0.54%	0.00%	35.2bp (0.0bp)	RELA/MA0107.1/Jaspar(0.655) More Information | Similar Motifs Found
14	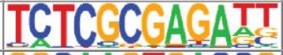	1e-20	-4.664e+01	1.68%	0.19%	57.1bp (57.0bp)	GFX(?)/Promoter/Homer(0.981) More Information | Similar Motifs Found
15	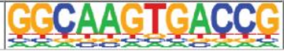	1e-17	-3.943e+01	0.49%	0.01%	1.0bp (0.0bp)	SD0002.1_at_AC_acceptor/Jaspar(0.718) More Information | Similar Motifs Found

B

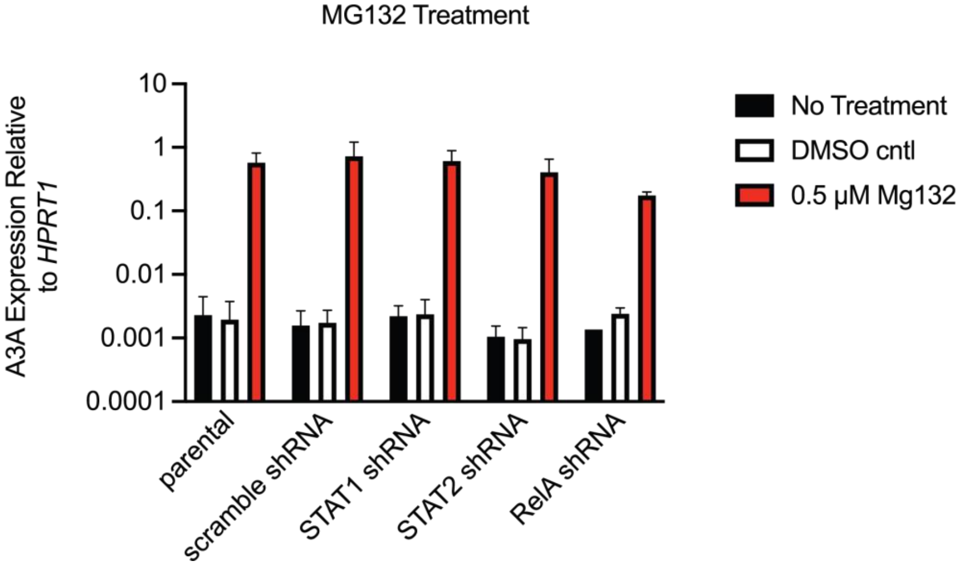

Supplemental Figure 2: Potential transcription factors regulating *APOBEC3A* induction upon proteasome inhibition. (A) Top 15 transcription factor binding sites enriched in promoter regions for transcripts with greater than or equal to 2-fold change

in abundance (measured by csRNA-seq and identified with HOMER (1)) from MDA-MB-453 cells treated with 0.5 μM MG132 compared to DMSO. (B) MG132 treatment of shRNA MDA-MB-453 lines. shRNA constructs targeting STAT1, STAT2, and RelA in MDA-MB-453 cells were treated with 0.5 μM MG132 for 24 hours. A non-targeting (scramble) shRNA and a DMSO treatment were included as controls. Expression of A3A was measured after treatment via qRT-PCR. A3A increased >100-fold in parental, scramble and each knockdown line in the presence of MG132.

Supplemental Figure 3

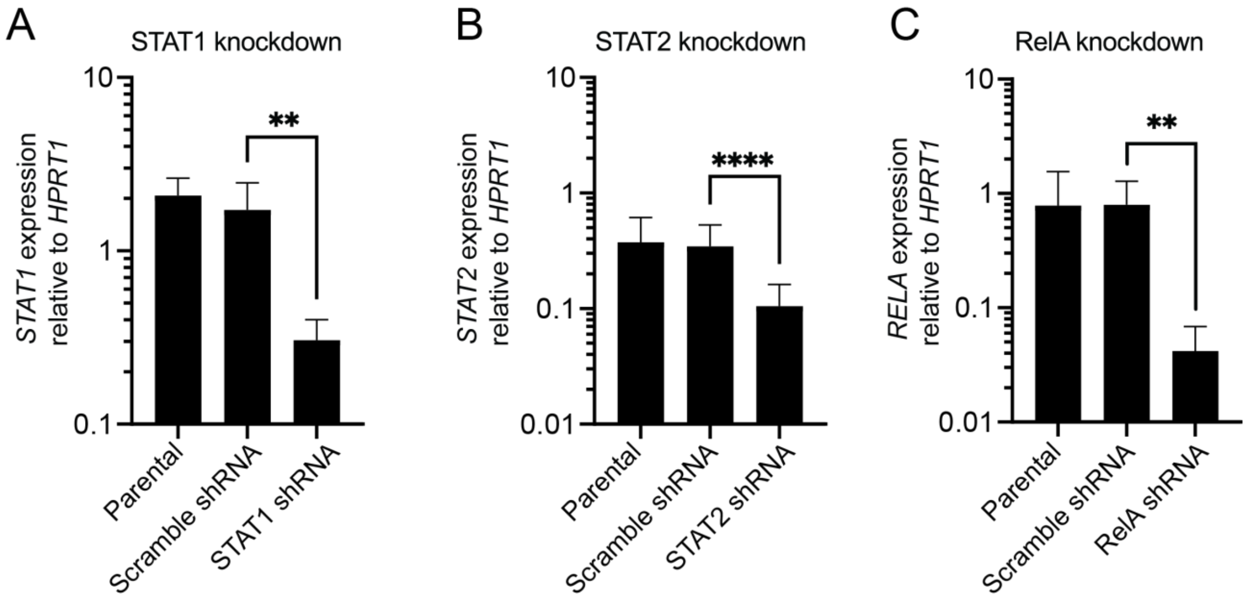

Supplemental Figure 3: qRT-PCR validation of transcription factor shRNA knockdowns. shRNA constructs targeting STAT1, STAT2, and RelA were transduced with lentivirus into MDA-MB-453 cells. A non-targeting (scramble) shRNA was included as a control. Expression of each gene was measured via qRT-PCR and fold reduction was calculated. (A) STAT1 expression was reduced 5-fold, (B) STAT2 expression was reduced 3.5-fold, and (C) RelA expression was reduced 19-fold. Comparisons made by ratio paired t test. ** = p value ≤ 0.01, **** = p value ≤ 0.0001,

Supplemental Figure 4

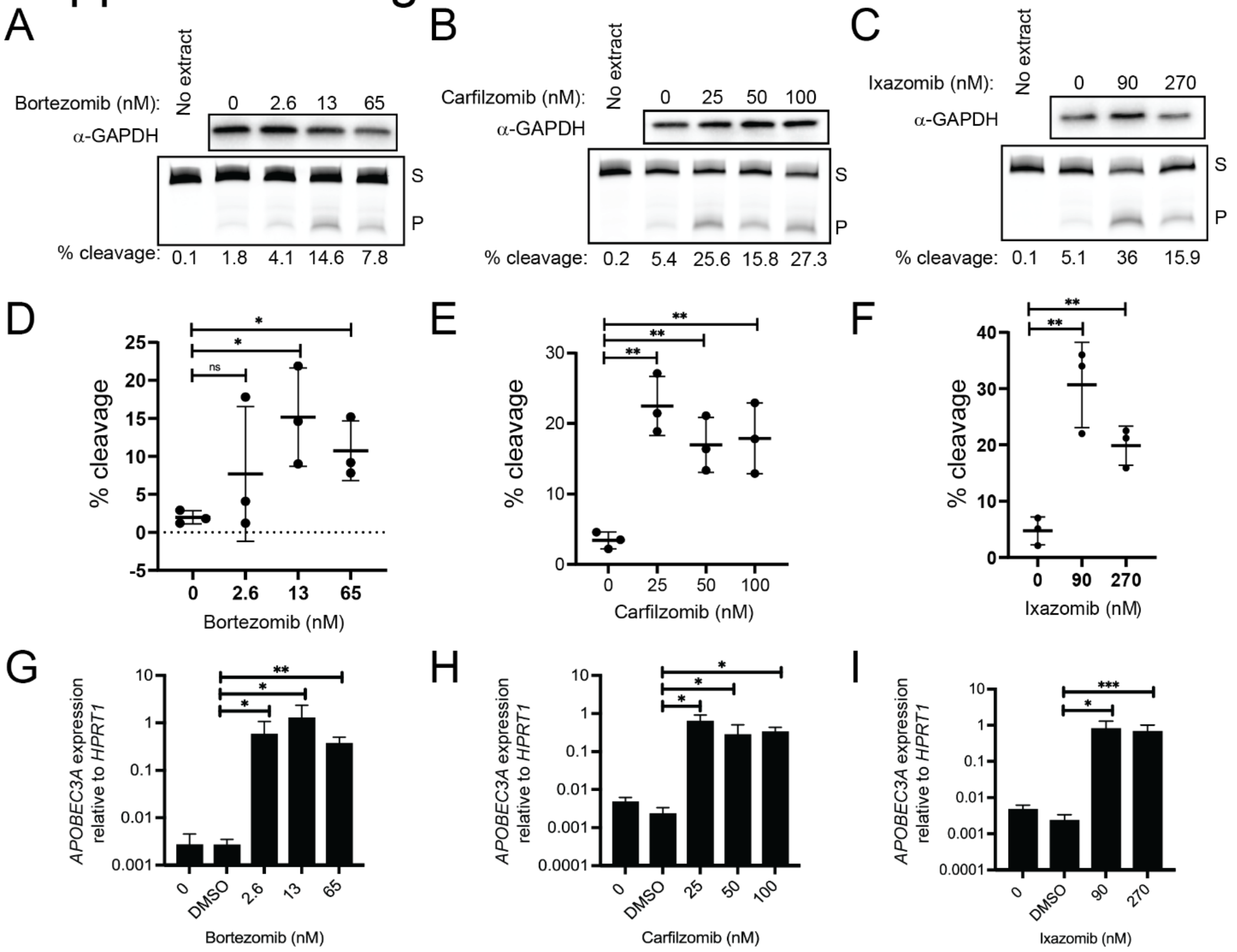

Supplemental Figure 4: Other proteasome inhibitors increase A3A in MDA-MB-453. (A,B,C) Representative images of western blot GAPDH analysis and denaturing gel for cytidine deaminase assay with percent cleavage shown for (A) bortezomib, (B) carfilzomib, and (C) ixazomib. Quantification of percent cleavage for all replicates (n=3) of bortezomib (D), carfilzomib (E), and ixazomib (F) treated cells from activity assays. Significant changes in activity were observed after treatments with each drug tested, as represented by * denoting the p value of unpaired t test results. Plots for (D, E, F) show the mean of all replicates, error bars represent one standard deviation. (G) Bortezomib at concentrations 2.6, 13, and 65 nM increased A3A transcript significantly. (H)

Carfilzomib at concentrations 25, 50, and 100 nM increased A3A significantly. (I) Ixazomib at concentrations 90 and 270 nM increased A3A transcript significantly. S = substrate, P= product. * = p value ≤ 0.05, ** = p value ≤ 0.01, *** = p value ≤ 0.001, n.s. = non-significant with p value > 0.05.

Supplemental Figure 5

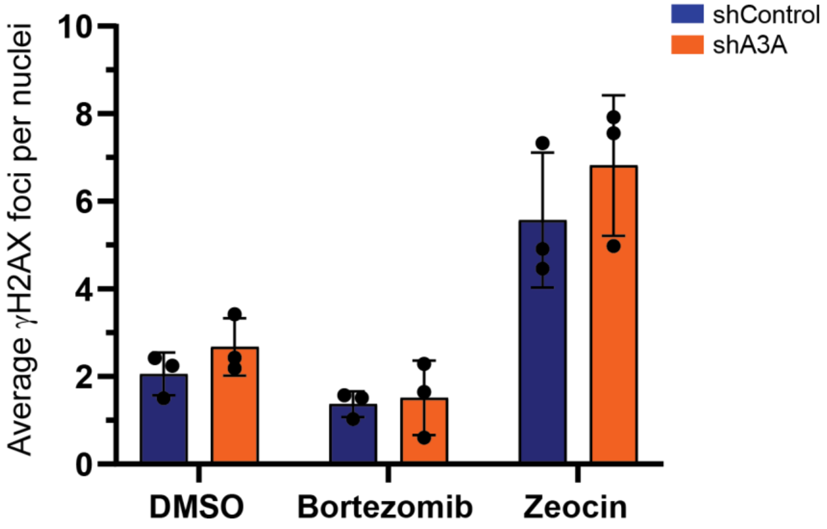

Supplemental Figure 5: Lack of A3A-mediated DSB formation in BT474 cells. The number of γH2AX foci per nuclei in BT474 cells transduced to express either control shRNA (shControl; shown in blue) or A3A targeting shRNA (shA3A; shown in orange) for 3 replicates. Error bars indicate standard deviation.
